# Combination of hydrogel nanoparticles and proteomics to reveal secreted proteins associated with decidualization of human uterine stromal cells

**DOI:** 10.1186/1477-5956-9-50

**Published:** 2011-09-01

**Authors:** Sarah Paule, Katie Meehan, Adam Rainczuk, Andrew N Stephens, Guiying Nie

**Affiliations:** 1 Prince Henrys Institute of Medical Research, Clayton 3168 Australia

## Abstract

**Background:**

Identification of secreted proteins of low abundance is often limited by abundant and high molecular weight (MW) proteins. We have optimised a procedure to overcome this limitation.

**Results:**

Low MW proteins in the conditioned media of cultured cells were first captured using dual-size exclusion/affinity hydrogel nanoparticles and their identities were then revealed by proteomics.

**Conclusions:**

This technique enables the analysis of secreted proteins of cultured cells low MW and low abundance.

## Background

Secretory proteins are collectively referred as the secretome, representing a major class of proteins located on or within the extracellular matrix or exported into extracellular fluids [1]. Secretomes contain hundreds, possibly thousands (depending on cell type and condition) of small to medium sized (< 50 kDa) peptides and proteins that reflect the physiological, metabolic and disease-associated biological states [2]. Proteomic analysis of small and low abundant secreted proteins is challenging requiring multiple prior fractionation processes [3,4], with the inherent variability and the removal of proteins bound to their 'non-specific' binding partners [5,6]. A recent publication demonstrates that low molecular weight (MW) proteins can be captured from complex plasma proteins by dual size exclusion/affinity nanoparticles (SEAN) [7]. The selective capturing involves the protein-binding of low MW proteins to the cation core which is surrounded by a porous acrylamide shell [8]. The nanoparticle sequesters low MW peptides and proteins and excludes proteins > 20000 Da, thereby enriching low MW proteins.

During the secretory phase of the menstrual cycle in women, the uterine stromal cells undergo a differentiation process called decidualization which is critical for pregnancy success. A poor decidual response is associated with infertility and/or pregnancy related disorders. Decidual cells are highly secretory and secreted prolactin and insulin-like growth factors are used as decidualization markers [9-11]. Further identification of novel secreted proteins is critical in determining factors that are important for decidualization and pregnancy outcome. Primary stromal cells isolated from human endometrium can be induced for decidualization *in vitro*. To date, the identification of low abundant secreted proteins has been restricted.

In this study, we successfully combined SEAN and proteomics to reveal secreted proteins of primary human decidual cells. This is the first publication describing a detailed protocol to first enrich secreted low MW proteins from cultured cells using SEAN, then to reveal their identities by proteomics.

## Results and Discussion

### Precipitation and SEAN of cultured media confirms success by SELDI-TOF mass spectroscopy (MS)

**Figure 1:**
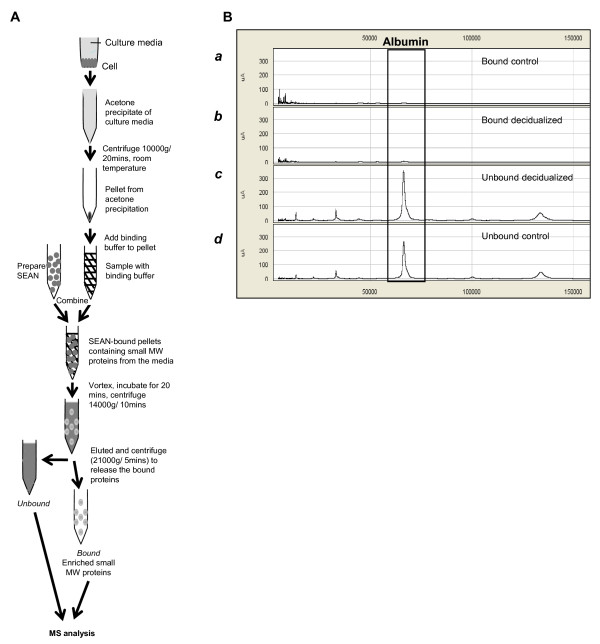
**Schematic diagram showing SEAN captures low MW proteins but leaves high MW proteins in culture media**. (***A***) The process of capturing low MW proteins from cultured media using SEAN. (***B***) Representative SELDI-ToF profiles shows the removal of albumin in SEAN-captured (control and decidualized) media samples. Albumin was absent in SEAN-bound (***a ***and ***b***) but present in SEAN-unbound proteins (***c ***and ***d***). Proteins over the range of 0-150 000 m/z are shown.

**Figure 2:**
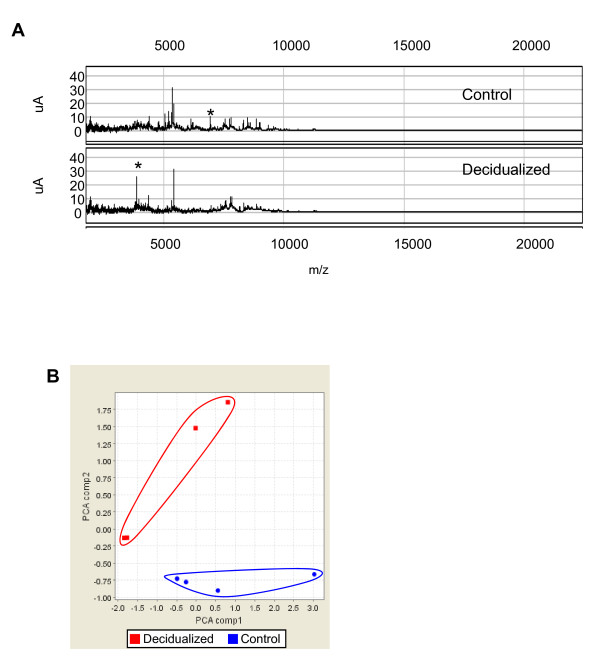
**Comparison of SEAN-bound proteins from control and decidualized cell media**. (***A***) Representative SELDI-ToF profiles shows a clear difference between control and decidualized media over the range of 0-25 000 m/z. (***B***) Principle components analysis (PCA) of all significant protein peaks, revealing a dramatic separation between the two groups over 4 separate experiments.

Decidualized and control cultured media were subjected to SEAN to capture low MW proteins from the complex protein mixture (Figure 1A). This procedure was previously performed in plasma [7] and SELDI-ToF MS confirmed the success of SEAN in cultured media by comparing the MW of SEAN-bound (small MW, proteins bound) and unbound (large MW such as BSA) proteins. SELDI-ToF profile of SEAN-bound fractions (< 25 000 m/z) of control and decidualized media showed that large molecular MW proteins (> 25 000 m/z) including BSA were completely removed in the SEAN bound fraction in both control and decidualized cell media (Figure 1B). We next compared the SELDI-ToF profile of SEAN-bound fractions (< 25 000 m/z) of control and decidualized media (Figure 2A). A number of protein peaks were present in the decidualized fraction but were not present in control fraction (asterisks, Figure 2A). Furthermore, Principle Components Analysis (PCA) demonstrated a clear difference in the secretomes between decidual and control cells (Figure 2B). These confirmed that SEAN procedure removed large MW proteins and enriched small MW proteins for further MS analysis.

### Identification of SEAN-bound proteins of decidual and control media

**Figure 3:**
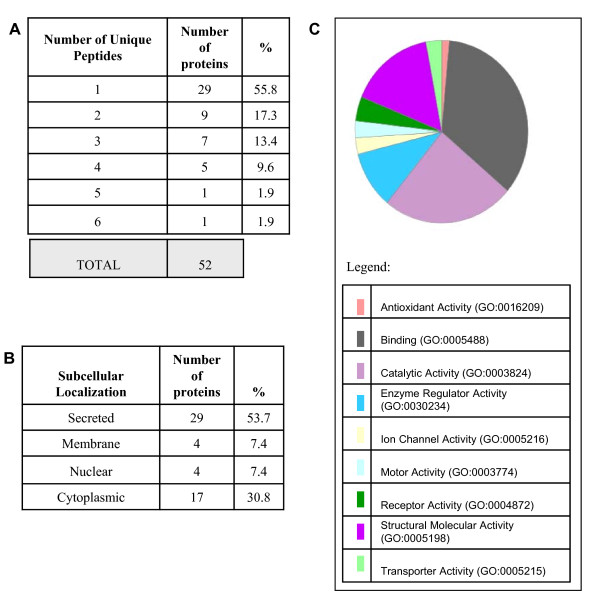
**Mass spectrometry analysis of secreted proteins from decidualized cells**. Stratification of proteins of interest was based on the number of peptides contributing to their identification (***A***), subcellular localization (***B***) and molecular function (***C***). (***A***) Representative SELDI-ToF profiles shows a clear difference between control and decidualized media over the range of 0-25 000 m/z. (***B***) Principle components analysis (PCA) of all significant protein peaks, revealing a dramatic separation between the two groups over 4 separate experiments.

We then analysed the SEAN-bound proteins of decidual and control media by proteomics. A total of 219 proteins were identified in the cultured media, 184 of which were from decidual media and 35 from control media (Additional file 1, Additional file 2 and Additional file 3). Of the 218 proteins from decidual cells, only 52 identified proteins were exclusively expressed in humans (Figure 3 and Additional file 3). Twenty-nine of these proteins (55.8%) contain a unique peptide fragment, the spectra of which were manually validated (Figure 3A). This is not surprising considering SEAN enriches proteins of small MW proteins. For the 52 identified proteins, Multiloc was applied to predict the subcellular localization (Figure 3B) [12]. Multiloc is based on the recognition of N-terminal targeting sequences, the overall amino acid composition, sequence motifs extracted from the database of nuclear localization signals [13] and the PROSITE database [14]. 53.8% were predicted to be secreted or potentially secreted, whilst 30.8% were localized to the cytoplasm, 7.7% to the nucleus or membrane (Figure 3B).

### Molecular functions of secreted decidual proteins

The PANTHER (Protein ANalysis THrough Evolutionary Relationships) classification system was used to classify the 52 decidual proteins according to their molecular functions (Figure 3C) [15,16]. A large percent (86.9%) were involved in binding, catalytic, enzyme regulatory and structural molecular activity. These are important for cell remodeling which is an essential component of decidualization. For example; the extracellular matrix protein, laminin subunit- beta 1 bind to integrins at the time of implantation thereby allowing blastocyst-endometrium interaction [17-19]. The identification of metalloproteinase inhibitor (MMP) 1 and HTRA1 in media of decidual cells highlights the power of SEAN. MMP inhibitors are known to be produced by decidual tissues throughout gestation [20-22]. Additional *in vitro *studies suggest that MMPs and their inhibitors must be finely regulated for implantation and pregnancy success [22,23].

## Conclusions

In summary, this study demonstrates that SEAN is a powerful fractionation strategy to remove a majority of serum containing proteins including albumin in the cell media to unmask low abundant secreted proteins. Proteomic analysis of SEAN enriched proteins, enables the identification of low abundant and low MW secreted proteins. The described protocol is based on decidual cell media and is equally applicable to other cell experiments.

## Methods

### Isolation and culture of human endometrial tissue

Ethical approval was obtained for all tissue collections from the Human Ethics Committee at Monash Medical Centre, Melbourne, Australia. Written and informed consent was obtained from all patients. Human uterine biopsies (days 8-24 of the menstrual cycle) was obtained from fertile women undergoing curettage. Samples were collected in Dulbecco's modified Eagle's medium/F12 (DMEM/F12; Sigma, St. Louis, MO) and processed within 24 hr. Human endometrial stromal cells (HESC) were isolated by enzymatic digestion and filtration as previously described [24]. Cells were decidualized with 8-bromoadenosine cAMP (500 μM), estradiol (E_2_; 10^-8 ^M) and medroxyprogesterone acetate (MPA; 10^-7 ^M) (all from Sigma) for 96 hours in DMEM/F12 containing 0.1% BSA. HESC treated with vehicle served as controls. At the end of the 96 hours, culture media was collected, aliquoted and stored at -80°C until analysis. The success of decidualization was confirmed by a significant increase in prolactin secretion (data not shown). Four biological replicates were conducted and pooled (4 controls and 4 decidual) for SEAN and proteomic analysis.

### Precipitation and SEAN of cultured media

Media from successfully decidualized and control stromal cells were thawed rapidly to room temperature and SEAN experiment was performed as described with modifications [7]. Precipitation and SEAN procedures were undertaken at room temperature unless suggested otherwise. Samples were precipitated with acetone in the dark overnight, and centrifuged (10 000 g/20 mins; Beckman JS7.5 rotor, NSW, Australia). The protein pellet was dried (maximum of 10 mins) and resuspended in 2 ml of binding buffer (MES 10 mM, NaCl 10 mM, pH 6). Precipitated proteins were solubilized in 2 ml binding buffer, mixed with the washed SEAN pellets, briefly vortexed and incubated for 20 mins. Following centrifugation (14 000 × g/10 mins), the pellet was vigorously resuspended and washed three times in 2 ml binding buffer containing 0.1% (w/v) n-Octyl β-D-glucopyranoside (OGP) (Sigma), and then three times in 2 ml of ice-cold distilled, deionized water. Low MW proteins remained bound to SEAN pellet, whilst high MW proteins remained unbound. The SEAN pellets were incubated with 200 μl elution buffer [80% acetonitrile (v/v; Merck, Darmstadt, Germany), 0.1%trifluoroacetic acid (TFA) v/v (Sigma)] for 5 mins and centrifuged at 21 000 g/10 mins (Eppendorf 5415 rotor, Hamburg, Germany) to recover the bound proteins. This procedure was performed twice to maximize recovery.

### SELDI-ToF MS

Proteins fractions from SEAN-bound and unbound proteins were concentrated at 60°C in a centrifugal concentrator (Eppendorf) to ~10 μl volume, and applied to Gold ProteinChip Array (BioRad, Hercules, CA) and analyzed using a ProteinChip Reader Series 4000 Personal Edition (BioRad) in positive ion mode. Spectra were acquired in the range 1 500-25 000 Da or 25 000-140 000 Da using an average of 2150 laser shots, 1800 nJ laser intensity and 800 MHz sampling rate. External calibration of the ProteinChip Reader was performed using the all-in protein/peptide standard (BioRad). The mass spectra were analyzed using Ciphergen Express Client version 3.0.6 software. Baseline subtraction and normalization on total ion current were performed for all spectra within a single experiment prior to comparative analyses.

### Identification of SEAN-bound proteins by MS

Proteins extracted from the SEAN were diluted with 40 μl of 100 mM ammonium bicarbonate (Sigma) and reduced with 5 mM DTT (Sigma) at 56°C for 30 mins. Samples were incubated with 55 μl of 50 mM ammonium bicarbonate and 25 mM of iodoacetamide (Sigma) for 30 mins at room temperature to alkylate thiol groups. Trypsin (protein: trypsin, 50:1, Promega, Madison, WI) was added and samples were digested overnight at 37°C in a humidified chamber. The peptide mixture was fractionated by nanoflow reversed-phase (RP) liquid chromatography using a 1200 series Capillary HPLC (Agilent Technologies, Santa Clara, CA) online equipped with a nanoAcquity C18 150150 × 1.0 mm i.d. column (Waters, Milford, MA), at 0.5 μl/min flow rate at 45°C in a linear gradient from 100% solvent A (0.1% v/v formic acid) to solvent B (0.1% v/v aqueous formic acid, 60% acetonitrile). Mass spectra were acquired using LTQ-Orbitrap mass spectrometer (Thermo Fisher Scientific, Waltham, MA) for automated MS/MS. Data dependent MS was analysed by acquiring one FTMS scan followed by MS2 on the top five most intense ions. Dynamic exclusion was enabled at repeat count 1, exclusion list size 500, exclusion duration 180 s, and exclusion mass width +/- 1.5 m/z. Collision induced dissociation was performed by setting the ion isolation width at 2 m/z, normalized collision energy at 35%, activation Q at 0.25 and an activation time at 30 ms.

Acquired spectra were searched against combinations of UniProtKB_SwissProt (release version 13.3), Uniprot_SwissProt (release version 13.3), UniProtKB_TrEMBL (release version 40.3) and ipi.HUMAN (release version 3.59) using Phenyx (Geneva Bioinformatics, Switzerland, http://phenyx.vital-it.ch/docs/pwi/PWITOC.html). Reverse databases for the above were searched to calculate false discovery rates. Search restrictions were carbamidomethylation (CAM-fixed modification), oxidation of methionine (variable modification), a parent ion mass tolerance of 20 ppm and 3 missed tryptic cleavage site. Precursor ion mass tolerance was set at 1.5 Da plus an allowance for one ^13^C. Each sample was analyzed by three separate injections and subsequent mass spectral analysis.

## Abbreviations

MW: Molecular Weight; HESC: Human endometrial stromal cells; cAMP: 8-bromoadenosine cAMP; E_2_: estradiol; MPA: medroxyprogesterone acetate; MMP: metalloproteinase inhibitor; PANTHER: Protein ANalysis THrough Evolutionary Relationships

## Competing interests

The authors declare that they have no competing interests.

## Authors' contributions

SP and KM contributed equally in experiments and the preparation of this manuscript. AR provided his expertise regarding SEAN. AS participated in the design of the study. GN conceived the study, participated in its design and coordination and aided in drafting the manuscript. All authors read and approved the final manuscript.

## Supplementary Material

Additional file 1 **Table S1**. Secretome of proteins from control media captured and identified by mass spectrometry. Click here for file

Additional file 2 **Table S2**. Secretome proteins from decidualized media by SEAN and identified by mass spectroscopy that did not meet inclusion criteria. Click here for file

Additional file 3 **Table S3**. Secretome proteins captured by SEAN and identified by mass spectrometry that met the inclusion criteria. The numbers listed in rows indicate the number of distinct peptides identified by mass spectrometry from the media of decidualized cells. Click here for file
