# Supplementary material for: Combination of hydrogel nanoparticles and proteomics to reveal secreted proteins associated with decidualization of human uterine stromal cells
Source: Proteome Sci. 2011 Sep 1;9:50. doi: 10.1186/1477-5956-9-50 (PMC3184050; doi:10.1186/1477-5956-9-50)
Supplement: Additional file 1 — Table S1. Secretome of proteins from control media captured and identified by mass spectrometry. [file 1477-5956-9-50-S1.PDF]

Additional file 1. Secretome of proteins from control media captured and identified by mass spectrometry.

	Accession Number	Protein(s) inferred	Mol Wt (kDa)	Number of Unique Peptides	Sequence Coverage	Position in sequence	Peptide Sequence	Precursor m/z	Charge observed	Calculated mass (M+H)	Mascot score	Mascot Expect value
1	P39656	sp|P39656|Dolichyl-diphosphooligosaccharide--protein glycosyltransferase 48 kDa subunit	50	1	4.61%	255 - 275	(R)VIFSGSLDFFSDSFFNSAVQK(A)	1171.57	2	2342.13	84.1	5.4E-05
2	P81644	sp|P81644|Apolipoprotein A-II	11	1	16.00%	79 - 94	(K)AGTDLLNFLSSFIDPK(K)	869.46	2	1737.91	120.0	1.7E-08
3	Q9BTM1	sp|Q9BTM1|Histone H2A.J	14	1	30.30%	53 - 72	(K)AAVLEYLTAEILELAGNAAR(D)	1044.57	2	2088.13	60.4	0.0151
						53 - 72	(K)AAVLEYLTAEILELAGNAAR(D)	1044.57	2	2088.13	53.2	0.0792
4	Q71U36	sp|Q71U36|Tubulin alpha-1A chain	50	4	17.10%	327 - 336	(K)DVNAAIATIK(T)	508.29	2	1015.58	44.6	0.536
						230 - 243	(R)LISQIVSSITASLR(F)	744.44	2	1487.88	61.9	0.00477
						125 - 156	(K)LADQcTGLQGFLVFHSFGGGTGSGFTSLLMER(L)	1130.88	3	3390.63	74.8	0.00046
						125 - 156	(K)LADQcTGLQGFLVFHSFGGGTGSGFTSLLmER(L)	1136.21	3	3406.62	68.3	0.00177
						244 - 264	(R)FDGALNVDLTEFQTNLVPYPR(I)	1205.11	2	2409.21	91.3	1.3E-05
5	P28800	sp|P28800|Alpha-2-antiplasmin	54	2	7.11%	152 - 162	(R)LcQDLGPGAFR(L)	617.31	2	1233.61	61.1	0.0091
						344 - 367	(K)YQLDLVATLSQLGLQELFQAPDLR(G)	1366.24	2	2731.47	69.0	0.0019

	Accession Number	Protein(s) inferred	Mol Wt (kDa)	Number of Unique Peptides	Sequence Coverage	Position in sequence	Peptide Sequence	Precursor m/z	Charge observed	Calculated mass (M+H)	Mascot score	Mascot Expect value
6	P12763	sp|P12763|Alpha-2-HS-glycoprotein	38	4	21.70%	121 - 131	(K)QDGQFSVLFTK(C)	635.33	2	1269.65	62.4	0.0069
						313 - 333	(R)HTFSGVASVESSSGEAFHVGK(T)	707.34	3	2120.00	70.6	0.00095
						29 - 50	(K)EPAcDDPDTEQAALAAVDYINK(H)	1203.54	2	2406.08	93.6	1.9E-06
						188 - 211	(R)AQFVPLPVSVSVEFAVAATDcIAK(E)	1260.16	2	2519.32	106.0	4.8E-07
7	Q99715	sp|Q99715|Collagen alpha-1(XII) chain	333	11	5.62%	461 - 468	(R)AFLEVLVK(S)	459.79	2	918.57	41.6	0.446
						1663 - 1672	(K)ITEVTSEGFR(G)	569.79	2	1138.57	56.9	0.0251
						2385 - 2395	(K)ALALGALQNIR(Y)	570.35	2	1139.69	83.1	2.2E-05
						1261 - 1272	(K)SLLQAVANLPYK(G)	658.88	2	1316.76	61.2	0.0107
						1954 - 1965	(R)WDPAPGPVLQYR(V)	699.86	2	1398.72	50.3	0.126
						1381 - 1396	(K)GPGDLEAPSNLVISER(T)	827.42	2	1653.85	52.9	0.0811
						500 - 513	(K)VEDIIEAINTFPYR(G)	840.44	2	1679.86	61.2	0.0138
						158 - 176	(K)YILDFIAALVSAFDIGEEK(T)	1057.56	2	2114.11	73.4	0.00085
						1130 - 1148	(R)LGELVVGPYDNTVVLEELR(A)	1058.07	2	2115.13	71.0	0.00138
						2057 - 2076	(R)IIYSPTVGDPIDEYTTVPGR(R)	1097.06	2	2193.11	58.4	0.0262
						2492 - 2522	(K)IEDNLITFVcETATSScPLIYLDGYTSPGFK(M)	1171.23	3	3511.67	70.5	0.00098

	Accession Number	Protein(s) inferred	Mol Wt (kDa)	Number of Unique Peptides	Sequence Coverage	Position in sequence	Peptide Sequence	Precursor m/z	Charge observed	Calculated mass (M+H)	Mascot score	Mascot Expect value
8	P06703	sp|P06703|Protein S100-A6	10	1	18.90%	2 - 18	(M)acPLDQAIGLLVAIFHK(Y)	954.52	2	1908.04	61.4	0.0104
9	P08123	sp|P08123|Collagen alpha-2(I) chain	129	4	5.05%	1308 - 1319	(R)FTYTVLVDGcSK(K)	695.34	2	1389.67	70.2	0.00093
						1193 - 1205	(K)VYcDFSTGETcIR(A)	804.35	2	1607.68	69.0	0.0002
						1141 - 1156	(K)SLNNQIETLLTPEGSR(K)	886.46	2	1771.92	51.2	0.126
						1339 - 1366	(R)LPFLDIAPLDIGGADQEFFVDIGPVcFK(-)	1547.28	2	3093.57	84.3	6.7E-05
10	P15497	sp|P15497|Apolipoprotein A-I	30	4	18.90%	249 - 260	(K)VSILAAIDEASK(K)	608.84	2	1216.68	62.4	0.0107
						36 - 46	(K)DFATVYVEAIK(D)	628.33	2	1255.66	51.5	0.0869
						51 - 63	(R)DYVAQFEASALGK(Q)	699.85	2	1398.69	54.5	0.0406
						69 - 82	(K)LLDNWDTLASTLSK(V)	788.92	2	1576.82	76.2	0.00044
11	P06576	sp|P06576|ATP synthase subunit beta, mitochondrial	43	1	3.96%	218 - 233	(R)DKEGQDVLLFIDNIFR(F)	961.49	2	1922.00	82.9	8.9E-05

	Accession Number	Protein(s) inferred	Mol Wt (kDa)	Number of Unique Peptides	Sequence Coverage	Position in sequence	Peptide Sequence	Precursor m/z	Charge observed	Calculated mass (M+H)	Mascot score	Mascot Expect value
12	P08758	sp|P08758|Annexin A5	35	5	25.00%	277 - 285	(R)SEIDLFNIR(K)	553.80	2	1106.58	44.5	0.601
						30 - 45	(K)GLGTDEESILTLLTSR(S)	852.95	2	1704.90	78.5	0.00026
						246 - 260	(R)SIPAYLAETLYYAMK(G)	867.45	2	1733.88	45.6	0.489
						162 - 186	(R)DPDAGIDEAQVEQDAQALFQAGELK(W)	886.76	3	2658.25	56.1	0.0262
						213 - 227	(K)YMTISGFQIEETIDR(E)	901.93	2	1802.86	66.5	0.00234
13	Q16777	sp|Q16777|Histone H2A type 2-C	13	1	40.00%	44 - 72	(R)VGAGAPVYMAAVLEYLTAEILELAGNAAR(D)	1467.28	2	2933.54	94.3	6.3E-06
						44 - 72	(R)VGAGAPVYmAAVLEYLTAEILELAGNAAR(D)	1475.27	2	2949.54	70.4	0.00162
14	Q99878	sp|Q99878|Histone H2A type 1-J	13	1	37.50%	101 - 119	(K)VTIAQGGVLPNIQAVLLPK(K)	644.39	3	1931.17	49.6	0.0199

	Accession Number	Protein(s) inferred	Mol Wt (kDa)	Number of Unique Peptides	Sequence Coverage	Position in sequence	Peptide Sequence	Precursor m/z	Charge observed	Calculated mass (M+H)	Mascot score	Mascot Expect value
15	P12111	sp|P12111|Collagen alpha-3(VI) chain	343	13	8.06%	1128 - 1143	(R)ITEGVPQLLIVLTADR(S)	580.01	3	1738.01	43.3	0.288
						2423 - 2441	(R)DVVLSIVNDLTIAESNcPR(G)	705.70	3	2115.08	46.9	0.362
						1258 - 1270	(R)LVDYLDVGFDTTR(V)	757.38	2	1513.75	59.8	0.0132
						610 - 625	(R)AAPLQGMLPGLLAPLR(T)	809.48	2	1617.95	67.9	0.00104
						1404 - 1418	(K)LLTPITTLTSEQIQK(L)	843.49	2	1685.97	52.4	0.0629
						1425 - 1456	(R)YPPPAVESDAADIVFLIDSSEGVRPDGFAHIR(D)	860.93	4	3440.71	45.7	0.467
						1381 - 1395	(K)ISLSPEYVFSVSTFR(E)	866.45	2	1731.90	45.8	0.467
						936 - 951	(R)IEDGVLQFLVLLVAGR(S)	871.52	2	1742.02	79.8	7.6E-05
						977 - 1001	(K)NADPAELEQIVLSPAFILAAESLPK(I)	879.48	3	2636.42	54.9	0.0489
						475 - 505	(R)LEIGQDLIQVAVAQYADTVRPEFYFNTHPTK(R)	891.71	4	3563.82	45.4	0.5
						1475 - 1491	(R)VGVVQFSNDVFPEFYLK(T)	994.51	2	1988.02	52.5	0.0998
						2714 - 2734	(R)ALGSAIEYTIENVFESAPNPR(D)	1139.57	2	2278.14	57.9	0.0288
						2008 - 2027	(R)LNLLDLDYELAEQLDNIAEK(A)	1166.60	2	2332.19	99.5	2.1E-06

	Accession Number	Protein(s) inferred	Mol Wt (kDa)	Number of Unique Peptides	Sequence Coverage	Position in sequence	Peptide Sequence	Precursor m/z	Charge observed	Calculated mass (M+H)	Mascot score	Mascot Expect value
16	P12109	sp|P12109|Collagen alpha-1(VI) chain	108	2	3.02%	791 - 803	(K)ENYAELLEDAFLK(N)	777.89	2	1554.77	70.3	0.00123
						223 - 240	(R)DAEEAISQTIDTIVDMIK(N)	996.50	2	1991.99	74.3	0.00059
17	P01966	sp|P01966|Hemoglobin subunit alpha	15	2	19.00%	129 - 140	(K)FLANVSTVLTSK(Y)	640.37	2	1279.73	66.0	0.00275
						18 - 32	(K)VGGHAAEYGAEALER(M)	765.37	2	1529.73	57.5	0.0162
18	P34955	sp|P34955|Alpha-1-antiproteinase	46	2	7.69%	197 - 213	(K)VLDPNTVFALVNYISFK(G)	647.36	3	1940.05	52.5	0.0811
						323 - 337	(K)SVLGDVGITEVFSDR(A)	797.41	2	1593.81	54.5	0.0629
19	P84243	sp|P84243|Histone H3.3	15	1	23.50%	85 - 116	(R)FQSAAIGALQEASEAYLVGLFEDTNLcAIHAK(R)	1146.57	3	3437.71	66.7	0.00354
20	A5D7R6	tr|A5D7R6|ITIH2 protein	106	3	6.45%	476 - 487	(R)IYGNQDTSVQLK(K)	683.35	2	1365.70	44.3	0.601
						380 - 394	(K)IQPSGGTNINEALLR(A)	791.93	2	1582.86	63.7	0.00644
						538 - 571	(K)LGQLQSIITATSANAELVLETLAEmDGLEDFLSK(D)	1212.96	3	3636.86	88.6	2.5E-05

	Accession Number	Protein(s) inferred	Mol Wt (kDa)	Number of Unique Peptides	Sequence Coverage	Position in sequence	Peptide Sequence	Precursor m/z	Charge observed	Calculated mass (M+H)	Mascot score	Mascot Expect value
21	P02751	sp|P02751|Fibronectin	262	15	10.10%	2231 - 2244	(K)LLcQcLGFGSGHFR(C)	551.27	3	1651.78	54.8	0.0346
						2165 - 2176	(R)GATYNIIVEALK(D)	646.37	2	1291.73	44.0	0.587
						912 - 922	(R)DLQFVEVTDVK(V)	646.84	2	1292.67	61.9	0.0112
						1650 - 1661	(K)IAWESPQGQVSR(Y)	679.35	2	1357.69	48.6	0.166
						1637 - 1649	(K)GLAFTDVDVDSIK(I)	690.36	2	1379.71	82.6	8.9E-05
						670 - 694	(K)GLKPGVVYEGQLISIQQYGHQEVTR(F)	700.63	4	2799.48	61.6	0.0123
						2150 - 2164	(R)VPGTSTSATLTGLTR(G)	731.40	2	1461.79	80.1	0.00018
						1867 - 1880	(R)SYTITGLQPGTDYK(I)	772.38	2	1543.76	64.3	0.00435
						117 - 130	(K)DSmIWDcTcIGAGR(G)	829.34	2	1657.68	59.9	0.00072
						923 - 938	(K)VTImWTPPESAVTGYR(V)	912.45	2	1823.90	52.2	0.091
						1892 - 1910	(R)SSPVVIDASTAIDAPSNLR(F)	957.00	2	1913.00	86.4	4.2E-05
						1285 - 1301	(R)VTWAPPPSIDLTNFLVR(Y)	963.53	2	1926.05	46.2	0.346
						1435 - 1452	(R)EESPLLIGQQSTVSDVPR(D)	978.01	2	1955.01	60.3	0.0162
						959 - 976	(R)NTFAEVTGLSPGVTYYFK(V)	997.50	2	1993.99	62.7	0.0091
						1453 - 1476	(R)DLEVVAATPTSLLISWDAPAVTVR(Y)	1262.69	2	2524.37	94.0	5.9E-06

	Accession Number	Protein(s) inferred	Mol Wt (kDa)	Number of Unique Peptides	Sequence Coverage	Position in sequence	Peptide Sequence	Precursor m/z	Charge observed	Calculated mass (M+H)	Mascot score	Mascot Expect value
22	XP_875097	ref|XP_875097|PREDICTED: similar to complement component 4A	193	13	12.80%	80 - 88	(K)VDFSLSSDR(D)	513.25	2	1025.49	49.7	0.0975
						492 - 501	(K)VGETLNLNLR(A)	564.82	2	1128.64	76.0	0.00032
						924 - 936	(R)GSFDFPVGDAISK(I)	670.33	2	1339.65	57.9	0.0208
						313 - 324	(K)LVDGQcQISLQK(A)	694.86	2	1388.72	77.6	0.0003
						1484 - 1504	(K)VGLSGmAIADITLLSGFHALR(A)	720.06	3	2158.17	73.6	0.00069
						604 - 621	(R)ALVALGAVDTALYAVGGK(S)	844.98	2	1688.96	112.0	7.2E-08
						737 - 750	(R)EPFLSccQFAESLR(K)	872.39	2	1743.78	58.9	0.00561
						89 - 103	(R)DFILLNVPIPQEQAR(V)	876.99	2	1752.97	82.6	7.4E-05
						1584 - 1600	(K)LLSTLcSADVcQcAEGK(C)	956.43	2	1911.86	88.4	6.7E-06
						764 - 782	(R)AMELLQEEDLIEEDDIPVR(S)	1129.05	2	2257.09	69.9	0.00132
						987 - 1012	(R)VTASDPLEALGSEGALSPGGLASLLR(L)	1241.16	2	2481.32	66.1	0.00416
						198 - 222	(R)EVFAPSSIFQDNFLIPDISAPGTWK(I)	1390.20	2	2779.40	48.9	0.239
						253 - 277	(K)IIPENPYILTTPGFLSDIQVIIQAR(Y)	1406.29	2	2811.57	61.8	0.00574

	Accession Number	Protein(s) inferred	Mol Wt (kDa)	Number of Unique Peptides	Sequence Coverage	Position in sequence	Peptide Sequence	Precursor m/z	Charge observed	Calculated mass (M+H)	Mascot score	Mascot Expect value
23	P20930	sp|P20930|Filaggrin	435	1	0.37%	2 - 16	(M)sTLLENIFAIINLFK(Q)	889.51	2	1778.01	58.7	0.0135
						2 - 16	(M)sTLLENIFAIINLFK(Q)	889.51	2	1778.01	47.3	0.186
24	P62805	sp|P62805|Histone H4	24	4	22.10%	47 - 56	(R)ISGLIYEETR(G)	590.81	2	1180.62	64.4	0.00548
						81 - 92	(K)TVTAMDVVYALK(R)	655.86	2	1310.70	67.1	0.00288
						25 - 36	(R)DNIQGITKPAIR(R)	663.38	2	1325.75	52.4	0.0574
						81 - 92	(K)TVTAmDVVYALK(R)	663.85	2	1326.70	66.4	0.00315
						155 - 169	(K)AMGIMNSFVNDIFER(M)	872.41	2	1743.82	75.1	0.00026
						155 - 169	(K)AmGIMNSFVNDIFER(M)	880.41	2	1759.81	58.9	0.0091
						155 - 169	(K)AmGImNSFVNDIFER(M)	888.41	2	1775.81	55.4	0.0162
25	P16104	sp|P16104|Histone H2A.x	15	2	36.40%	97 - 119	(K)LLGGVTIAQGGVLPNIQAVLLPK(K)	1136.19	2	2271.38	56.2	0.00245
						44 - 72	(R)VGAGAPVYLAAVLEYLTAEILELAGNAAR(D)	1458.30	2	2915.59	124.0	4.6E-09

	Accession Number	Protein(s) inferred	Mol Wt (kDa)	Number of Unique Peptides	Sequence Coverage	Position in sequence	Peptide Sequence	Precursor m/z	Charge observed	Calculated mass (M+H)	Mascot score	Mascot Expect value
26	P07996	sp|P07996|Thrombospondin-1	129	9	10.90%	87 - 95	(K)GFLLLASLR(Q)	495.31	2	989.61	70.1	0.00048
						155 - 164	(K)SITLFVQEDR(A)	604.32	2	1207.63	52.5	0.0931
						125 - 137	(K)AGTLDLSLTVQGK(Q)	651.87	2	1302.73	49.9	0.186
						518 - 529	(R)LcNNPTPQFGGK(D)	666.82	2	1332.64	43.7	0.371
						1042 - 1054	(K)QVTQSYWDTNPTR(A)	798.38	2	1595.75	72.8	0.00044
						202 - 216	(K)GGVNDNFQGVLQNVR(F)	808.91	2	1616.81	74.5	0.00055
						530 - 543	(K)DcVGDVTENQIcNK(Q)	826.36	2	1651.71	86.9	3.5E-06
						21 - 41	(R)IPESGGDNSVFDIFELTGAAR(K)	1098.03	2	2195.06	95.3	4E-06
						265 - 285	(K)DLQAIcGIScDELSSMVLELR(G)	1205.08	2	2409.15	89.2	1.4E-05
27	Q2KIS7	sp|Q2KIS7|Tetranectin	22	4	29.70%	63 - 73	(K)EQQALQTVcLK(G)	659.35	2	1317.68	59.6	0.0186
						49 - 62	(K)TQLDSLAQEVALLK(E)	764.93	2	1528.86	67.3	0.00269
						156 - 169	(K)NWETEITAQPDGGK(V)	773.36	2	1545.72	51.9	0.0435
						102 - 122	(R)GGTLGTPQTGSENDALYEYLR(Q)	1121.53	2	2242.06	80.1	0.00011

	Accession Number	Protein(s) inferred	Mol Wt (kDa)	Number of Unique Peptides	Sequence Coverage	Position in sequence	Peptide Sequence	Precursor m/z	Charge observed	Calculated mass (M+H)	Mascot score	Mascot Expect value
28	P02452	sp|P02452|Collagen alpha-1(I) chain	138	11	14.10%	575 - 586	(R)GQAGVmGFPGPK(G)	581.29	2	1161.57	60.3	0.00998
						1237 - 1246	(K)SLSQQIENIR(S)	594.32	2	1187.64	69.1	0.00204
						1406 - 1424	(R)FTYSVTVDGcTSHTGAWGK(T)	691.98	3	2073.93	43.0	0.229
						1387 - 1399	(K)ALLLQGSNEIEIR(A)	728.41	2	1455.82	46.7	0.269
						68 - 79	(K)VLcDDVIcDETK(N)	733.83	2	1466.65	57.5	0.00674
						1067 - 1084	(R)GETGPAGPAGPVGPVGAR(G)	773.90	2	1546.80	57.0	0.0362
						613 - 631	(K)DGEAGAQGPPGPAGPAGER(G)	845.89	2	1690.78	50.2	0.0615
						1289 - 1310	(K)VFcNmETGETcVYPTQPSVAQK(N)	854.71	3	2562.13	45.0	0.0953
						1271 - 1288	(K)SGEYWIDPNQGcNLDAIK(V)	1040.48	2	2079.94	62.1	0.0033
						80 - 114	(K)NcPGAEVPEGEccPVcPDGSESPTDQETTGVEGPK(G)	1249.51	3	3746.52	75.4	7.4E-06
						1437 - 1464	(R)LPIIDVAPLDVGAPDQEFGFDVGPVcFL(-)	1500.76	2	3000.51	62.3	0.0104

	Accession Number	Protein(s) inferred	Mol Wt (kDa)	Number of Unique Peptides	Sequence Coverage	Position in sequence	Peptide Sequence	Precursor m/z	Charge observed	Calculated mass (M+H)	Mascot score	Mascot Expect value
29	Q15582	sp|Q15582|Transforming growth factor-beta-induced protein ig-h3	74	7	22.40%	571 - 588	(K)YHIGDEILVSGGIGALVR(L)	623.68	3	1869.02	44.0	0.561
						77 - 90	(K)STVISYEccPGYEK(V)	846.87	2	1692.73	68.3	0.0003
						534 - 548	(R)EGVYTVFAPTNEAFR(A)	850.92	2	1700.83	47.5	0.204
						515 - 533	(R)FSMLVAAIQSAGLTETLNR(E)	1011.54	2	2022.07	72.7	0.00109
						378 - 396	(K)TLFELAAESDVSTAIDLFR(Q)	1049.54	2	2098.07	78.1	0.00028
						128 - 172	(K)LRPEmEGPGSFTIFAPSNEAWASLPAEVLDSLVSNVNIELLNALR(Y)	1221.63	4	4883.49	60.9	0.00889
						235 - 257	(K)VISTITNNIQQIIEIEDTFETLR(A)	1346.21	2	2690.43	75.3	0.00054
30	P68431	sp|P68431|Histone H3.1	15	1	23.50%	85 - 116	(R)FQSSAVmALQEAcEAYLVGLFEDTNLcAIHAK(R)	1201.57	3	3602.70	76.8	0.0002
31	Q15063	sp|Q15063|Periostin	93	5	10.40%	252 - 265	(R)AAAITSDILEALGR(D)	700.89	2	1400.78	78.6	0.00023
						509 - 522	(R)FSTFLSLLEAADLK(E)	777.93	2	1554.84	56.6	0.0308
						38 - 53	(R)DQGPNVcALQQILGTK(K)	871.45	2	1741.89	64.1	0.00644
						523 - 542	(K)ELLTQPGDWTLFVPTNDAFK(G)	1146.58	2	2292.16	56.2	0.0435
						229 - 251	(R)VLTQIGTSIQDFIEAEDDLSSFR(A)	1292.64	2	2584.28	107.0	3.3E-07

	Accession Number	Protein(s) inferred	Mol Wt (kDa)	Number of Unique Peptides	Sequence Coverage	Position in sequence	Peptide Sequence	Precursor m/z	Charge observed	Calculated mass (M+H)	Mascot score	Mascot Expect value
32	Q00610	sp|Q00610|Clathrin heavy chain 1	191	4	4.24%	994 - 1010	(K)AFmTADLPNELIELLEK(I)	982.01	2	1963.01	45.7	0.512
						812 - 830	(R)LPVVIGGLLDVDcSEDVIK(N)	1021.05	2	2041.09	72.1	0.00104
						1294 - 1311	(R)GYFEELITMLEAALGLER(A)	1028.03	2	2055.05	61.9	0.0112
						1294 - 1311	(R)GYFEELITmLEAALGLER(A)	1036.02	2	2071.04	57.2	0.0323
						1546 - 1562	(K)DTELAEELLQWFLQEEK(R)	1061.02	2	2121.04	54.5	0.0512
33	Q3SZQ8	tr|Q3SZQ8|Endopin 2	47	4	14.60%	322 - 330	(K)DILSQLGIK(K)	493.80	2	986.59	45.1	0.536
						332 - 347	(K)IFTSDADFSGITDDHK(L)	590.27	3	1768.80	42.1	0.33
						153 - 168	(K)DAEAFYASEVLSTNFK(D)	896.42	2	1791.84	59.5	0.00953
						192 - 211	(K)LFNDLDVLTNLILLNYIFFK(A)	1214.68	2	2428.35	60.0	0.0102
34	P02081	sp|P02081|Hemoglobin fetal subunit beta	15	9	64.10%	66 - 75	(K)VLDSFcEGLK(Q)	584.29	2	1167.57	54.9	0.0338
						132 - 143	(K)VVTGVANALAHR(Y)	604.35	2	1207.69	83.4	4.9E-05
						104 - 115	(R)LLGNVLVVVLAR(R)	633.42	2	1265.83	78.5	6.7E-06
						30 - 39	(R)LLVVYPWTQR(F)	637.87	2	1274.73	47.2	0.281
						65 - 75	(K)KVLDSFcEGLK(Q)	648.34	2	1295.67	50.8	0.129
						17 - 29	(K)VKVDEVGGEALGR(L)	664.86	2	1328.72	56.9	0.0301
						117 - 131	(R)FGSEFSPELQASFQK(V)	851.41	2	1701.81	78.2	0.00014
						116 - 131	(R)RFGSEFSPELQASFQK(V)	929.46	2	1857.91	57.8	0.0223
						40 - 58	(R)FFESFGDLSSADAILGNPK(V)	1007.99	2	2014.98	86.2	2.9E-05

	Accession Number	Protein(s) inferred	Mol Wt (kDa)	Number of Unique Peptides	Sequence Coverage	Position in sequence	Peptide Sequence	Precursor m/z	Charge observed	Calculated mass (M+H)	Mascot score	Mascot Expect value
35	P02769	sp|P02769|Serum albumin	69	17	37.60%	360 - 371	(R)RHPEYAVSVLLR(L)	480.61	3	1439.81	57.1	0.0204
						66 - 75	(K)LVNELTEFAK(T)	582.32	2	1163.63	44.9	0.536
						581 - 597	(K)ccAADDKEAcFAVEGPK(L)	643.27	3	1927.80	47.3	0.0169
						319 - 336	(K)DAIPENLPPLTADFAEDK(D)	652.66	3	1955.96	53.6	0.0644
						569 - 580	(K)TVmENFVAFVDK(C)	708.35	2	1415.69	55.5	0.0269
						286 - 297	(K)YIcDNQDTISSK(L)	722.83	2	1443.64	42.1	0.245
						421 - 433	(K)LGEYGFQNALIVR(Y)	740.89	2	1479.80	54.2	0.0644
						375 - 386	(K)EYEATLEEccAK(D)	751.81	2	1502.61	64.4	0.0003
						438 - 451	(K)VPQVSTPTLVEVSR(S)	756.42	2	1511.84	44.4	0.426
						347 - 359	(K)DAFLGSFLYEYSR(R)	784.38	2	1567.74	73.2	0.00047
						437 - 451	(R)KVPQVSTPTLVEVSR(S)	820.47	2	1639.94	82.5	5.5E-05
						45 - 65	(K)GLVLIAFSQYLQQcPFDEHVK(L)	831.43	3	2492.27	61.1	0.0151
						469 - 482	(R)MPcTEDYLSLILNR(L)	862.92	2	1724.84	66.0	0.00301
						469 - 482	(R)mPcTEDYLSLILNR(L)	870.92	2	1740.83	94.9	3.3E-06
						184 - 197	(K)YNGVFQEccQAEDK(G)	874.36	2	1747.71	78.1	8.9E-06
						267 - 280	(K)EccHGDLLEcADDR(A)	875.34	2	1749.66	55.6	0.00034
						169 - 183	(R)HPYFYAPELLYYANK(Y)	944.97	2	1888.93	43.5	0.629
						529 - 544	(K)LFTFHADIcTLPDTEK(Q)	954.46	2	1907.92	60.3	0.0115
