# Supplementary material for: Combination of hydrogel nanoparticles and proteomics to reveal secreted proteins associated with decidualization of human uterine stromal cells
Source: Proteome Sci. 2011 Sep 1;9:50. doi: 10.1186/1477-5956-9-50 (PMC3184050; doi:10.1186/1477-5956-9-50)
Supplement: Additional file 2 — Table S2. Secretome proteins from decidualized media by SEAN and identified by mass spectroscopy that did not meet inclusion criteria. [file 1477-5956-9-50-S2.PDF]

Additional file 2. Secretome proteins from decidualized media by SEAN and identified by mass spectroscopy that did not meet inclusion criteria

	Accession Number	Protein(s) inferred	Mol Wt (kDa)	Number of Unique Peptides	Sequence Coverage	Position in sequence	Peptide Sequence	Precursor m/z	Charge observed	Calculated mass (M+H)	Mascot score	Mascot Expect value
1	Q99988	sp|Q99988|Growth/differentiation factor 15	34	2	8.44%	95 - 104	(R)LGSGGHLHLR(I)	523.80	2	1046.59	46.2	0.406
						288 - 303	(K)TDTGVSLQTYD	870.44	2	1739.87	80.5	0.00014
2	P62491	sp|P62491|Ras-related protein Rab-11A	24	1	5.09%	62 - 72	(K)AQIWDTAGQER(Y)	637.81	2	1274.61	58.0	0.0148
3	Q58D62	sp|Q58D62|Fetuin-B	42	2	5.17%	358 - 366	(K)VVVLPFPSK(E)	493.31	2	985.61	46.8	0.177
						168 - 178	(R)FMETATESLAK(Y)	614.30	2	1227.59	45.7	0.262
4	P21333	sp|P21333|Filamin-A	280	1	0.30%	302 - 309	(R)AEFTVETR(S)	476.74	2	952.47	47.3	0.269
5	P15497	sp|P15497|Apolipoprotein A-I	30	5	14.70%	249 - 260	(K)VSILAAIDEASK(K)	608.85	2	1216.68	81.2	0.00014
						36 - 46	(K)DFATVYVEAIK(D)	628.34	2	1255.66	65.6	0.00338
						249 - 261	(K)VSILAAIDEASKK(L)	672.89	2	1344.77	45.7	0.323
						51 - 63	(R)DYVAQFEASALGK(Q)	699.85	2	1398.69	76.9	0.00025
						34 - 46	(R)VKDFATVYVEAIK(D)	741.92	2	1482.82	47.3	0.213

	Accession Number	Protein(s) inferred	Mol Wt (kDa)	Number of Unique Peptides	Sequence Coverage	Position in sequence	Peptide Sequence	Precursor m/z	Charge observed	Calculated mass (M+H)	Mascot score	Mascot Expect value
6	P14678	sp|P14678|Small nuclear ribonucleoprotein-associated proteins B and B'	24	1	3.33%	66 - 73	(R)VLGLVLLR(G)	441.81	2	882.61	55.4	0.00109
7	P62991	sp|P62991|Ubiquitin	15	1	11.60%	12 - 27	(K)TITLEVEPSDTIENVK(A)	894.47	2	1787.93	52.5	0.12
8	P27797	sp|P27797|Calreticulin	48	2	6.24%	25 - 36	(K)EQFLDGDGWTSR(W)	705.82	2	1410.63	45.1	0.155
						74 - 87	(R)FYALSASFEPFSNK(G)	804.40	2	1607.77	79.0	0.00018
9	P61204	sp|P61204|ADP-ribosylation factor 3	20	1	11.60%	118 - 127	(R)DAVLLVFANK(Q)	545.32	2	1089.63	54.0	0.0512
10	P10145	sp|P10145|Interleukin-8	11	1	16.20%	54 - 69	(R)VIESGPHcANTEIIVK(L)	883.96	2	1766.91	66.7	0.00406
11	P07195	sp|P07195|L-lactate dehydrogenase B chain	36	2	8.38%	159 - 170	(R)VIGSGcNLDSAR(F)	624.81	2	1248.60	56.0	0.0256
						8 - 23	(K)LIAPVAEEEATVPNNK(I)	847.96	2	1694.90	50.0	0.169

	Accession Number	Protein(s) inferred	Mol Wt (kDa)	Number of Unique Peptides	Sequence Coverage	Position in sequence	Peptide Sequence	Precursor m/z	Charge observed	Calculated mass (M+H)	Mascot score	Mascot Expect value
12	B2FQP3	tr|B2FQP3|Conserved hypothetical exported protein Tax_Id=522373 [Stenotrophomonas maltophilia]	44	5	19.70%	405 - 417	(R)VLVNTKATIAAFR(-)	702.43	2	1403.84	72.4	0.00031
						266 - 279	(R)GTSDGYMDSIHTSR(N)	763.83	2	1526.65	76.8	5.4E-05
						379 - 404	(R)LNYWSNPNISYNGIPMGNASTADNQR(V)	966.45	3	2897.33	67.2	0.00155
						337 - 355	(R)HDIATDSSTSPYAYGHGYR(Y)	1049.47	2	2097.93	99.1	5E-07
						337 - 365	(R)HDIATDSSTSPYAYGHGYRYEPATGTGWR(T)	1072.82	3	3216.44	79.8	5.2E-05
13	O46375	sp|O46375|Transthyretin	15	1	8.84%	56 - 68	(K)AADETWEPFASGK(T)	704.83	2	1408.64	52.8	0.0323
14	P00749	sp|P00749|Urokinase-type plasminogen activator	48	1	2.78%	44 - 55	(K)YFSNIHWcNcPK(K)	813.36	2	1625.70	49.6	0.0315
15	P07741	sp|P07741|Adenine phosphoribosyltransferase	19	1	6.11%	2 - 12	(M)aDSELQLVEQR(I)	665.34	2	1329.67	50.5	0.102
16	P10599	sp|P10599|Thioredoxin	11	1	12.40%	9 - 21	(K)TAFQEALDAAGDK(L)	668.82	2	1336.64	91.4	7.6E-06

	Accession Number	Protein(s) inferred	Mol Wt (kDa)	Number of Unique Peptides	Sequence Coverage	Position in sequence	Peptide Sequence	Precursor m/z	Charge observed	Calculated mass (M+H)	Mascot score	Mascot Expect value
17	P10909	sp|P10909|Clusterin	52	4	12.50%	326 - 336	(R)ELDESLQVAER (L)	644.82	2	1288.64	48.5	0.158
						183 - 194	(R)ASSIIDELFQDR (F)	697.35	2	1393.70	64.6	0.00524
						307 - 322	(R)EILSVDcSTNNP SQAK(L)	881.92	2	1762.83	69.3	0.00098
						409 - 425	(K)LFDSDPITVTVP VEVSR(K)	937.50	2	1873.99	66.0	0.005
18	P05106	sp|P05106|Integrin beta-3	87	1	2.03%	73 - 88	(K)DNcAPESIEFPV SEAR(V)	910.91	2	1820.81	53.6	0.0177
19	Q12797	sp|Q12797|Aspartyl/asparaginy l beta-hydroxylase	85	1	2.37%	87 - 104	(K)LGIYDADGDGD FDVDDAK(V)	950.91	2	1900.81	85.2	4.7E-06
20	P10619	sp|P10619|Lysosomal protective protein	54	1	2.71%	253 - 265	(K)DLEcVTNLQEV AR(I)	773.88	2	1546.75	74.8	0.0004

	Accession Number	Protein(s) inferred	Mol Wt (kDa)	Number of Unique Peptides	Sequence Coverage	Position in sequence	Peptide Sequence	Precursor m/z	Charge observed	Calculated mass (M+H)	Mascot score	Mascot Expect value
21	P12763	sp|P12763|Alpha-2-HS-glycoprotein	38	7	30.10%	58 - 67	(K)HTLNQIDSVK(V)	577.81	2	1154.62	49.4	0.132
						121 - 131	(K)QDGQFSVLFTK(C)	635.33	2	1269.65	58.2	0.0186
						132 - 143	(K)cDSSPDSAEDVR(K)	669.27	2	1337.53	56.3	0.00098
						313 - 333	(R)HTFSGVASVESSSGEAFHVGK(T)	707.34	3	2120.00	68.2	0.00166
						334 - 348	(K)TPIVGQPSIPGGPVR(L)	737.92	2	1474.84	58.4	0.019
						104 - 120	(R)QQTQHAVEGDcDIHVLK(Q)	989.47	2	1977.95	61.0	0.00849
						29 - 50	(K)EPAcDDPDTEQAALAAVDYINK(H)	1203.54	2	2406.08	108.0	6.7E-08
22	P13639	sp|P13639|Elongation factor 2	95	3	4.08%	639 - 647	(K)YEWDVAEAR(K)	569.76	2	1138.52	54.5	0.0195
						560 - 571	(K)DLEEDHAcIPIK(K)	720.35	2	1439.68	54.9	0.0308
						581 - 594	(R)ETVSEESNVLcLSK(S)	797.88	2	1594.76	86.6	2.5E-05

	Accession Number	Protein(s) inferred	Mol Wt (kDa)	Number of Unique Peptides	Sequence Coverage	Position in sequence	Peptide Sequence	Precursor m/z	Charge observed	Calculated mass (M+H)	Mascot score	Mascot Expect value
23	A5D7S8	tr|A5D7S8|FBLN1 protein	77	2	5.10%	167 - 182	(K)VSEIEEEQEDPYLNDR(C)	982.94	2	1964.87	64.7	0.0012
						147 - 166	(K)GQETADFAPGDGGDLQETAK(V)	1003.95	2	2006.89	72.8	0.0002
24	O15173	sp|O15173|Membrane-associated progesterone receptor component 2	23	1	8.52%	79 - 97	(R)GLGAGAGAGEESPATSLPR(M)	849.42	2	1697.85	63.4	0.00629
25	O15460	sp|O15460|Prolyl 4-hydroxylase subunit alpha-2	60	1	2.62%	384 - 397	(K)SSWLEEDDDPVVAR(V)	809.37	2	1617.74	64.3	0.00213
26	O14818	sp|O14818|Proteasome subunit alpha type-7	27	1	6.05%	175 - 189	(K)NYTDEAIETDDLTIK(L)	870.91	2	1740.82	110.0	8.7E-08
27	Q9TTE1	sp|Q9TTE1|Serpin A3-1	46	4	12.90%	395 - 404	(K)DTQSIIFLGK(V)	561.31	2	1121.62	56.4	0.0346
						87 - 98	(R)GSTLTEILEGLK(F)	630.86	2	1260.71	64.7	0.00561
						197 - 208	(R)TELVLVNYIYFK(A)	751.42	2	1501.83	70.0	0.00166
						147 - 165	(K)FIEDAQVLYSSEAFPTNFR(D)	1117.54	2	2234.08	56.0	0.0323
28	P17697	sp|P17697|Clusterin	51	1	5.01%	63 - 72	(K)LLLSSLEEAK(K)	551.82	2	1102.64	57.2	0.0294
29	P17690	sp|P17690|Beta-2-glycoprotein 1	38	1	6.67%	228 - 250	(K)DTATFGcHETYSLDGPEEVEcSK(F)	878.03	3	2632.08	42.5	0.0308
30	P63027	sp|P63027|Vesicle-associated membrane protein 2	12	1	14.70%	67 - 83	(R)ADALQAGASQFETSAAK(L)	833.41	2	1665.81	86.4	2.9E-05

	Accession Number	Protein(s) inferred	Mol Wt (kDa)	Number of Unique Peptides	Sequence Coverage	Position in sequence	Peptide Sequence	Precursor m/z	Charge observed	Calculated mass (M+H)	Mascot score	Mascot Expect value
31	P02461	sp|P02461|Collagen alpha-1(III) chain	138	3	2.73%	1140 - 1150	(R)GPVGPSGPPGK(D)	475.26	2	949.51	53.2	0.0757
						1274 - 1286	(K)SGEYWVDPNQGcK(L)	770.33	2	1539.65	57.9	0.00275
						1240 - 1255	(K)SVNGQIESLISPDGSR(K)	830.41	2	1658.84	53.1	0.0601
32	P05362	sp|P05362|Intercellular adhesion molecule 1	57	2	4.32%	450 - 458	(R)DLEGTYLcR(A)	563.76	2	1126.52	43.5	0.275
						274 - 287	(K)ASVSVTAEDEGTQR(L)	725.34	2	1449.68	63.0	0.00406
33	P35232	sp|P35232|Prohibitin	29	2	8.09%	134 - 143	(R)FDAGELITQR(E)	575.30	2	1149.59	56.2	0.0371
						106 - 117	(R)IFTSIGEDYDER(V)	722.83	2	1444.66	59.4	0.00574
34	P60866	sp|P60866|40S ribosomal protein S20	13	1	9.24%	9 - 19	(K)TPVEPEVAIHR(I)	416.56	3	1247.67	64.7	0.00629
35	P08134	sp|P08134|Rho-related GTP-binding protein RhoC	21	1	8.81%	52 - 68	(K)QVELALWDTAGQEDYDR(L)	1004.96	2	2008.93	93.7	2.6E-06
36	P02545	sp|P02545|Lamin-A/C	74	6	9.79%	241 - 249	(R)LADALQELR(A)	514.79	2	1028.57	74.4	0.0004
						124 - 133	(K)EGDLIAAQAR(L	522.28	2	1043.55	65.8	0.00275
						63 - 72	(R)ITESEEVVSR(E)	574.79	2	1148.58	81.0	0.00012
						79 - 89	(K)AAYEAELGDAR(K)	583.28	2	1165.55	53.3	0.0371
						440 - 450	(R)VAVEEVDEEGK(F)	602.29	2	1203.57	76.7	0.00016
						12 - 25	(R)SGAQASSTPLSPTR(I)	680.35	2	1359.69	53.5	0.0601

	Accession Number	Protein(s) inferred	Mol Wt (kDa)	Number of Unique Peptides	Sequence Coverage	Position in sequence	Peptide Sequence	Precursor m/z	Charge observed	Calculated mass (M+H)	Mascot score	Mascot Expect value
37	P63261	sp|P63261|Actin, cytoplasmic 2	41	1	28.50%	2 - 18	(M)eEEIAALVIDNGSGmcK(A)	947.43	2	1893.86	74.2	0.00021
38	P18065	sp|P18065|Insulin-like growth factor-binding protein 2	35	1	3.96%	228 - 240	(R)TPcQQELDQVLER(I)	808.39	2	1615.78	80.1	9.5E-05
39	P62304	sp|P62304|Small nuclear ribonucleoprotein E	10	1	13.00%	81 - 92	(K)GDNITLLQSVSN(-)	630.83	2	1260.64	69.9	0.00155
40	O43399	sp|O43399|Tumor protein D54	22	1	7.77%	35 - 50	(R)TPAVEGLTEAEEEELR(A)	886.93	2	1772.86	112.0	6.7E-08
41	P27824	sp|P27824|Calnexin	67	1	1.69%	283 - 292	(R)KPEDWDERPK(I)	433.88	3	1299.63	41.0	0.74
42	O76061	sp|O76061|Stanniocalcin-2	33	1	3.97%	143 - 154	(K)HDLcAAAQENTR(V)	693.31	2	1385.62	60.6	0.00379
43	P23142	sp|P23142|Fibulin-1	77	2	4.69%	147 - 163	(K)SQETGDLDVGGLQETDK(I)	896.41	2	1791.83	94.8	2.1E-06
						164 - 179	(K)IIEVEEEQEDPYLNDR(C)	995.97	2	1990.92	77.9	0.00012
44	P22105	sp|P22105|Tenascin-X	464	1	0.26%	750 - 760	(R)VPSSASAYDQR(G)	590.78	2	1180.56	66.0	0.00208
45	P62701	sp|P62701|40S ribosomal protein S4, X isoform	29	1	4.94%	156 - 168	(K)VNDTIQIDLETGK(I)	723.38	2	1445.75	75.4	0.00048
46	Q9UNL2	sp|Q9UNL2|Translocon-associated protein subunit gamma	21	1	7.57%	9 - 22	(K)QQSEEDLLLQDFSR(N)	854.41	2	1707.82	96.2	2.7E-06
47	P61106	sp|P61106|Ras-related protein Rab-14	23	1	6.51%	157 - 170	(K)TGENVEDAFLEAAK(K)	747.36	2	1493.71	70.4	0.00089

	Accession Number	Protein(s) inferred	Mol Wt (kDa)	Number of Unique Peptides	Sequence Coverage	Position in sequence	Peptide Sequence	Precursor m/z	Charge observed	Calculated mass (M+H)	Mascot score	Mascot Expect value
48	Q9Y4K0	sp|Q9Y4K0|Lysyl oxidase homolog 2	86	1	1.55%	150 - 161	(K)HTEDVGVVcSDK(R)	673.31	2	1345.61	57.1	0.00774
49	Q9Y4L1	sp|Q9Y4L1|Hypoxia up-regulated protein 1	111	1	1.20%	159 - 170	(R)SLAEDFAEQPIK(D)	674.34	2	1347.68	55.2	0.0446
50	P00978	sp|P00978|Protein AMBP	39	1	4.26%	335 - 349	(K)EYcGIPGEADEELLR(F)	875.90	2	1750.80	54.4	0.0208
51	P62491	sp|P62491|Ras-related protein Rab-11A	24	2	9.26%	5 - 13	(R)DDEYDYLFK(V)	604.26	2	1207.52	39.6	0.213
						62 - 72	(K)AQIWDTAGQER(Y)	637.81	2	1274.61	63.9	0.0033
52	Q9UKU9	sp|Q9UKU9|Angiopoietin-related protein 2	57	1	2.43%	150 - 161	(R)DNALELSQLENR(I)	701.35	2	1401.70	71.0	0.00107
53	Q8IUX7	sp|Q8IUX7|Adipocyte enhancer-binding protein 1	130	2	2.85%	733 - 745	(R)YLSPDATVSTEVR(A)	719.36	2	1437.72	60.2	0.0138
						794 - 813	(R)GEDEDEVSEAQETPDHAIFR(W)	758.66	3	2273.98	48.5	0.0288
54	Q3SZ57	sp|Q3SZ57|Alpha-fetoprotein	68	3	5.08%	108 - 116	(K)YGLSDccSR(T)	559.22	2	1117.44	39.0	0.0548
						405 - 414	(K)YIQESQALAK(R)	575.81	2	1150.61	57.5	0.0281
						272 - 283	(K)GNVLEcLQDGER(V)	695.32	2	1389.64	70.0	0.00059
55	P09455	sp|P09455|Retinol-binding protein 1	15	1	8.89%	70 - 81	(K)EFEEDLTGIDDR(K)	719.82	2	1438.63	58.0	0.00512

	Accession Number	Protein(s) inferred	Mol Wt (kDa)	Number of Unique Peptides	Sequence Coverage	Position in sequence	Peptide Sequence	Precursor m/z	Charge observed	Calculated mass (M+H)	Mascot score	Mascot Expect value
56	P07996	sp|P07996|Thrombospondin-1	129	14	18.10%	102 - 109	(R)GTLLALER(K)	436.76	2	872.52	54.9	0.0536
						87 - 95	(K)GFLLLASLR(Q)	495.31	2	989.61	70.3	0.00046
						75 - 83	(K)FQDLVDAVR(A)	531.78	2	1062.56	51.7	0.123
						155 - 164	(K)SITLFVQEDR(A)	604.32	2	1207.63	51.8	0.115
						1131 - 1141	(K)IMADSGPIYDK(T)	605.29	2	1209.58	46.7	0.204
						518 - 529	(R)LcNNPTPQFGGK(D)	666.82	2	1332.64	48.6	0.12
						217 - 228	(R)FVFGTTPEDILR(N)	697.87	2	1394.73	46.3	0.397
						461 - 479	(R)LcNSPSPQmNGKPcEGEAR(E)	716.97	3	2147.93	33.7	0.574
						811 - 822	(R)DNcQYVYNVDQR(D)	787.34	2	1573.67	60.7	0.00151
						1042 - 1054	(K)QVTQSYWDTNPTR(A)	798.38	2	1595.75	69.8	0.00089
						202 - 216	(K)GGVNDNFQGVLQNVR(F)	808.91	2	1616.81	75.8	0.00041
						530 - 543	(K)DcVGDVTENQIcNK(Q)	826.36	2	1651.71	83.8	6.9E-06
						852 - 890	(R)IGDTcDNNQDIDEDGHQNNLDNcPYVPNANQADHDKDGK(G)	886.17	5	4425.83	57.7	0.00043
						823 - 851	(R)DTDMDGVGDQcDNcPLEHNPDQLDSDSDR(I)	1107.43	3	3320.27	68.8	1.1E-05
						823 - 851	(R)DTDmDGVGDQcDNcPLEHNPDQLDSDSDR(I)	1112.76	3	3336.26	60.1	7.6E-05

	Accession Number	Protein(s) inferred	Mol Wt (kDa)	Number of Unique Peptides	Sequence Coverage	Position in sequence	Peptide Sequence	Precursor m/z	Charge observed	Calculated mass (M+H)	Mascot score	Mascot Expect value
57	P11021	sp|P11021|78 kDa glucose-regulated protein	72	8	18.00%	50 - 60	(R)VEIIANDQGNR(I)	614.82	2	1228.63	51.3	0.0811
						622 - 633	(K)ELEEIVQPIISK(L)	699.40	2	1397.79	61.2	0.0083
						102 - 113	(R)TWNDPSVQQDIK(F)	715.85	2	1430.69	55.8	0.0251
						61 - 74	(R)ITPSYVAFTPEGER(L)	783.89	2	1566.78	52.1	0.0931
						353 - 367	(K)KSDIDEIVLVGGSTR(I)	794.93	2	1588.85	94.2	7.4E-06
						82 - 96	(K)NQLTSNPENTVFDAK(R)	839.41	2	1677.81	62.5	0.00629
						307 - 324	(R)IEIESFYEGEDFSETLTR(A)	1083.00	2	2164.99	86.4	1.5E-05
						634 - 654	(K)LYGSAGPPPTGEEDTAEKDEL(-)	1088.50	2	2175.99	79.1	7.4E-05
58	Q28107	sp|Q28107|Coagulation factor V	248	3	1.81%	1637 - 1646	(R)AEVDDVIQVR(F)	572.30	2	1143.60	82.7	6.2E-05
						1957 - 1969	(K)EVLLTGIQTQGAK(H)	679.39	2	1357.77	66.9	0.00315
						1587 - 1603	(K)FVQSDDVDYVPEDTVYK(K)	1009.96	2	2018.92	102.0	3.5E-07
59	P19338	sp|P19338|Nucleolin	76	2	3.24%	334 - 342	(K)NDLAVVDVR(I)	500.78	2	1000.54	53.9	0.0446
						349 - 362	(K)FGYVDFESAEDLEK(A)	824.87	2	1648.74	67.3	0.00074
60	Q02388	sp|Q02388|Collagen alpha-1(VII) chain	295	3	1.19%	152 - 162	(K)SQDLVDTAAQR(L)	602.30	2	1203.60	60.5	0.00998
						79 - 89	(R)FATVQYSDDPR(T)	649.80	2	1298.60	43.4	0.269
						237 - 249	(R)DLVLSEPSSQSLR(V)	715.88	2	1430.75	46.8	0.346

	Accession Number	Protein(s) inferred	Mol Wt (kDa)	Number of Unique Peptides	Sequence Coverage	Position in sequence	Peptide Sequence	Precursor m/z	Charge observed	Calculated mass (M+H)	Mascot score	Mascot Expect value
61	P23284	sp|P23284|Peptidyl-prolyl cis-trans isomerase B	23	4	21.30%	172 - 180	(K)VLEGMEVVR(K)	516.28	2	1031.56	56.9	0.0379
						99 - 109	(K)DFMIQGGDFTR(G)	643.80	2	1286.58	42.1	0.281
						72 - 84	(K)TVDNFVALATGEK(G)	682.86	2	1364.71	63.7	0.00723
						146 - 158	(K)DTNGSQFFITTVK(T)	729.86	2	1457.73	51.2	0.0757
62	A6QPP2	tr|A6QPP2|SERPIND1 protein	55	2	6.05%	365 - 377	(K)TLESQLTPQAVER(W)	736.39	2	1471.78	62.1	0.00953
						327 - 343	(K)GAFLAASDQELDcDVLR(L)	940.45	2	1879.89	90.8	7.4E-06
63	XP_583270	ref|XP_583270|PREDICTED: apolipoprotein A	516	1	0.24%	4046 - 4056	(R)VLELDDEVQIK(A)	650.85	2	1300.70	68.3	0.00234
64	NP_0011007554	ref|NP_0011007554|Angiotensinogen (serpin peptidase inhibitor, clade A, member 8)	52	4	13.40%	388 - 400	(K)ASYDLQDLLAQAK(L)	718.37	2	1435.74	64.3	0.00629
						237 - 250	(R)SLDLSTDPNLAAEK(I)	737.38	2	1473.74	62.0	0.00998
						179 - 201	(K)VLSSLQTIQGLLVAQGGASSQAR(L)	762.09	3	2284.26	65.3	0.00362
						76 - 90	(K)SSAVDEEALWEQLVR(A)	866.43	2	1731.86	98.9	1.7E-06
65	P05121	sp|P05121|Plasminogen activator inhibitor 1	45	1	2.24%	146 - 154	(K)QVDFSEVER(A)	554.77	2	1108.53	53.6	0.0379
66	Q32KY0	sp|Q32KY0|Apolipoprotein D	21	1	6.35%	165 - 176	(K)DILTSNNIEVEK(M)	687.86	2	1374.71	66.3	0.00379
67	O43169	sp|O43169|Cytochrome b5 type B	16	1	8.22%	13 - 24	(K)GQEVETSVTYYR(L)	716.34	2	1431.68	71.6	0.00049
68	P18085	sp|P18085|ADP-ribosylation factor 4	20	2	11.10%	118 - 127	(R)DAVLLLFANK(Q)	552.33	2	1103.65	56.5	0.0288
						100 - 109	(R)IQEVADELQK(M)	586.81	2	1172.62	45.9	0.346

	Accession Number	Protein(s) inferred	Mol Wt (kDa)	Number of Unique Peptides	Sequence Coverage	Position in sequence	Peptide Sequence	Precursor m/z	Charge observed	Calculated mass (M+H)	Mascot score	Mascot Expect value
69	P40616	sp|P40616|ADP-ribosylation factor-like protein 1	20	1	7.18%	105 - 117	(K)SELVAMLEEEE LR(K)	774.38	2	1547.76	69.7	0.00144
70	P02774	sp|P02774|Vitamin D-binding protein	52	1	3.59%	403 - 419	(K)GQELcADYSEN TFTEYK(K)	1027.93	2	2054.87	99.3	1.7E-07
71	P12111	sp|P12111|Collagen alpha-3(VI) chain	343	6	2.33%	875 - 885	(R)IAVAQYSDDVK(V)	604.81	2	1208.62	70.3	0.00135
						1271 - 1281	(R)VAVIQFSDDPK(V)	609.82	2	1218.64	61.0	0.0144
						1029 - 1041	(K)DVVFLLDGSEG VR(S)	703.37	2	1405.73	95.2	5.5E-06
						1348 - 1360	(K)SDDEVDDPAVE LK(Q)	716.33	2	1431.65	71.0	0.00034
						561 - 573	(K)SLDEISQPAQE LK(R)	729.38	2	1457.75	58.1	0.0218
						2994 - 3006	(R)EVQVFEITENS AK(L)	747.38	2	1493.75	55.8	0.0397
72	O00622	sp|O00622|Protein CYR61	42	1	3.41%	182 - 194	(K)ELGFDASEVEL TR(N)	733.36	2	1465.72	58.5	0.0182
73	P02751	sp|P02751|Fibronectin	262	5	2.89%	1117 - 1129	(K)LGVRPSQGGE APR(E)	441.91	3	1323.71	43.6	0.615
						370 - 379	(R)TFYScTTEGR(Q)	611.26	2	1221.52	41.8	0.117
						912 - 922	(R)DLQFVEVTDVK (V)	646.84	2	1292.67	63.6	0.00757
						84 - 100	(R)GFNcESKPEAE ETcFDK(Y)	683.28	3	2047.84	42.6	0.0338
						1435 - 1452	(R)EESPLLIGQQS TVSDVPR(D)	978.01	2	1955.01	62.7	0.00931
74	Q13765	sp|Q13765|Nascent polypeptide-associated complex subunit alpha	23	2	13.50%	114 - 127	(K)SPASDTYIVFG EAK(I)	742.87	2	1484.73	72.0	0.00071
						128 - 142	(K)IEDLSQQAQLA AAEK(F)	807.92	2	1614.83	73.9	0.00063

	Accession Number	Protein(s) inferred	Mol Wt (kDa)	Number of Unique Peptides	Sequence Coverage	Position in sequence	Peptide Sequence	Precursor m/z	Charge observed	Calculated mass (M+H)	Mascot score	Mascot Expect value
75	O14498	sp|O14498|Immunoglobulin superfamily containing leucine-rich repeat protein	45	1	2.57%	129 - 139	(K)mDSNELTFIPR(D)	669.82	2	1338.64	58.9	0.0132
76	P09211	sp|P09211|Glutathione S-transferase P	23	1	7.14%	31 - 45	(K)EEVVTVETWQEGSLK(A)	867.43	2	1733.86	88.6	2E-05
77	P62753	sp|P62753|40S ribosomal protein S6	28	1	6.02%	32 - 46	(R)mATEVAADALGEEWK(G)	818.88	2	1636.75	98.9	7.7E-07
78	P62805	sp|P62805|Histone H4	24	5	26.60%	25 - 36	(R)DNIQGITKPAIR(R)	442.59	3	1325.75	43.9	0.406
						69 - 78	(R)DAVTYTEHAK(R)	567.78	2	1134.54	43.8	0.371
						47 - 56	(R)ISGLIYEETR(G)	590.81	2	1180.62	67.2	0.00288
						81 - 92	(K)TVTAmDVVYALK(R)	663.85	2	1326.70	49.2	0.162
						155 - 169	(K)AmGImNSFVNDIFER(M)	888.41	2	1775.81	50.6	0.0477
79	P30049	sp|P30049|ATP synthase subunit delta, mitochondrial	17	1	8.33%	137 - 150	(K)AQAELVGTADEATR(A)	716.36	2	1431.71	89.7	1.3E-05
80	P60953-2	sp_vs|P60953-2|(CDC42)Isoform 2 of P60953.	21	2	12.00%	154 - 163	(K)YVEcSALTQK(G)	599.79	2	1198.58	46.3	0.199
						108 - 120	(K)TPFLLVGTQIDLR(D)	736.93	2	1472.85	45.7	0.354
81	P18669	sp|P18669|Phosphoglycerate mutase 1	28	1	6.30%	142 - 157	(R)YADLTEDQLPScESLK(D)	934.93	2	1868.86	75.1	0.00019
82	Q01518	sp|Q01518|Adenylyl cyclase-associated protein 1	51	1	3.79%	331 - 348	(R)VENQENVSNLVIEDTELK(Q)	1037.02	2	2073.04	93.4	7.7E-06
83	P04075	sp|P04075|Fructose-bisphosphate aldolase A	39	2	6.32%	61 - 69	(R)QLLLTADDR(V)	522.79	2	1044.57	54.5	0.0536
						29 - 42	(K)GILAADESTGSIAK(R)	666.85	2	1332.70	73.3	0.00083
84	P08253	sp|P08253|72 kDa type IV collagenase	73	1	1.82%	520 - 531	(K)IDAVYEAPQEEK(A)	696.34	2	1391.67	45.1	0.308

	Accession Number	Protein(s) inferred	Mol Wt (kDa)	Number of Unique Peptides	Sequence Coverage	Position in sequence	Peptide Sequence	Precursor m/z	Charge observed	Calculated mass (M+H)	Mascot score	
85	P47985	sp|P47985|Cytochrome b-c1 complex subunit Rieske, mitochondrial	29	1	5.11%	183 - 196	(K)EIEQEAAVELSQLR(D)	807.92	2	1614.83	67.5	0.00281
86	P21980	sp|P21980|Protein-glutamine gamma-glutamyltransferase 2	77	1	2.18%	635 - 649	(K)TVEIPDPVEAGEEVK(V)	806.41	2	1611.81	47.5	0.262
87	P10809	sp|P10809|60 kDa heat shock protein, mitochondrial	61	2	3.84%	421 - 429	(R)VTDALNATR(A)	480.76	2	960.51	50.4	0.155
						206 - 218	(K)TLNDELEIIEGMK(F)	752.88	2	1504.76	74.0	0.0006
88	P62826	sp|P62826|GTP-binding nuclear protein Ran	24	1	5.09%	61 - 71	(K)FNVWDTAGQEK(F)	647.81	2	1294.61	54.0	0.0308
89	A8E647	tr|A8E647|A2M protein	167	4	3.38%	72 - 81	(R)SLFTDVVAEK(D)	554.80	2	1108.59	57.2	0.0281
						1160 - 1170	(R)NALFcLDSAWK(S)	662.82	2	1324.64	44.3	0.371
						326 - 338	(K)IQEEGTEVELTGK(G)	716.86	2	1432.72	83.0	6.7E-05
						718 - 734	(R)DFVHFDDTSEPPTETVR(K)	996.45	2	1991.90	57.2	0.00889
90	P01137	sp|P01137|Transforming growth factor beta-1	44	1	3.33%	279 - 291	(R)ALDTNYcFSSTEK(N)	768.34	2	1535.67	85.9	6.7E-06
91	O46375	sp|O46375|Transthyretin	15	2	19.00%	56 - 68	(K)AADETWEPFASGK(T)	704.82	2	1408.64	59.0	0.00615
						69 - 83	(K)TSESGELHGLTTEDK(F)	802.38	2	1603.75	72.1	0.00045
92	P20339	sp|P20339|Ras-related protein Rab-5A	23	1	5.12%	71 - 81	(K)FEIWDTAGQER(Y)	676.32	2	1351.63	65.0	0.00229
93	P00749	sp|P00749|Urokinase-type plasminogen activator	48	1	2.32%	109 - 118	(R)SDALQLGLGK(H)	501.29	2	1001.56	57.7	0.0301
94	P23528	sp|P23528|Cofilin-1	18	2	13.90%	2 - 13	(M)aSGVAVSDGVIK(V)	572.81	2	1144.62	61.8	0.0109
						82 - 92	(R)YALYDATYETK(E)	669.32	2	1337.63	47.0	0.158

	Accession Number	Protein(s) inferred	Mol Wt (kDa)	Number of Unique Peptides	Sequence Coverage	Position in sequence	Peptide Sequence	Precursor m/z	Charge observed	Calculated mass (M+H)	Mascot score	Mascot Expect value
95	P69905	sp|P69905|Hemoglobin subunit alpha	15	1	8.45%	129 - 140	(K)FLASVSTVLTSK(Y)	626.86	2	1252.72	79.7	9.5E-05
96	P15880	sp|P15880|40S ribosomal protein S2	31	1	4.78%	90 - 103	(K)ESEIIDFFLGASLK(D)	784.92	2	1568.82	79.6	0.00017
97	P68104	sp|P68104|Elongation factor 1-alpha 1	50	3	11.30%	85 - 96	(K)YYVTIIDAPGHR(D)	468.91	3	1404.73	51.1	0.138
						256 - 266	(K)IGGIGTVPVGR(V)	513.31	2	1025.61	54.6	0.0234
						101 - 129	(K)NmITGTSQADcAVLIVAAGVGEFEAGISK(N)	975.82	3	2925.43	76.0	0.0004
98	Q3T004	sp|Q3T004|Serum amyloid P-component	25	1	7.14%	150 - 165	(K)IVLGQEQDSYGGGFDK(N)	856.91	2	1712.81	94.3	3.5E-06
99	Q9BUD6	sp|Q9BUD6|Spondin-2	35	1	6.34%	155 - 175	(R)IVPSPDWFVGVDSLDLcDGDR(W)	1181.55	2	2362.10	69.9	0.00089
100	P09871	sp|P09871|Complement C1s subcomponent	76	1	2.33%	265 - 280	(K)SNALDIIFQTDLTGQK(K)	882.46	2	1763.92	73.3	0.00085
101	Q9BTM1	sp|Q9BTM1|Histone H2A.J	14	1	30.30%	53 - 72	(K)AAVLEYLTAEILELAGNAAR(D)	1044.57	2	2088.13	60.4	0.0151
						53 - 72	(K)AAVLEYLTAEILELAGNAAR(D)	1044.57	2	2088.13	53.2	0.0792
102	P28800	sp|P28800|Alpha-2-antiplasmin	54	2	7.11%	152 - 162	(R)LcQDLGPGAFR(L)	617.31	2	1233.61	61.1	0.0091
						344 - 367	(K)YQLDLVATLSQLGLQELFQAPDLR(G)	1366.24	2	2731.47	69.0	0.0019
103	P05556	sp|P05556|Integrin beta-1	88	1	4.01%	445 - 476	(K)IRPLGFTEEVEVILQYIcEcEcQSEGIPESPK(C)	1270.61	3	3809.81	53.4	0.0467
104	P61009	sp|P61009|Signal peptidase complex subunit 3	20	1	12.80%	61 - 83	(R)SDLGFITFDITADLENIFDWNVK(Q)	1337.16	2	2673.31	78.4	0.00023

	Accession Number	Protein(s) inferred	Mol Wt (kDa)	Number of Unique Peptides	Sequence Coverage	Position in sequence	Peptide Sequence	Precursor m/z	Charge observed	Calculated mass (M+H)	Mascot score	Mascot Expect value
105	P06868	sp|P06868|Plasminogen	91	4	8.64%	646 - 656	(R)EQSVQEIPVSR(L)	636.33	2	1271.66	46.6	0.269
						553 - 573	(R)KLFDYcDVPQcESSFDcGKPK(V)	860.71	3	2580.12	47.4	0.0406
						289 - 305	(K)NYGGTVAVTESGHTcQR(W)	918.92	2	1836.83	70.5	0.00046
						697 - 717	(R)TEcYITGWGETQGTFGEGLLK(E)	1174.05	2	2347.09	112.0	5.2E-08
106	Q8SPP7	sp|Q8SPP7|Peptidoglycan recognition protein	21	4	35.30%	141 - 152	(R)AAQSLLAcGAAR(G)	594.81	2	1188.62	44.2	0.561
						50 - 69	(R)YVVVSHTAGSVcNTPAScQR(Q)	731.68	3	2193.02	57.7	0.0132
						166 - 179	(R)DVQQTLSPGDELYK(I)	796.89	2	1592.78	64.6	0.00467
						83 - 103	(R)GWcDVGYNFLIGEDGLVYEGR(G)	1210.05	2	2419.10	62.2	0.00354
107	P31949	sp|P31949|Protein S100-A11	11	1	15.20%	37 - 52	(K)TEFLSFMNTELAAFTK(N)	925.46	2	1849.90	59.8	0.0151
108	P78527	sp|P78527|DNA-dependent protein kinase catalytic subunit	469	3	1.16%	3800 - 3813	(R)LGLIEWLENTVTLK(D)	814.97	2	1628.93	58.2	0.019
						1838 - 1852	(R)EFFSTIVVDAIDVLK(S)	848.46	2	1695.92	44.1	0.548
						3129 - 3147	(K)LQSVQALTEIQEFISFISK(Q)	1091.09	2	2181.18	82.7	0.0001
109	Q16777	sp|Q16777|Histone H2A type 2-C	13	1	40.00%	44 - 72	(R)VGAGAPVYMAAVLEYLTAEILELAGNAAR(D)	1467.28	2	2933.54	94.3	6.3E-06
						44 - 72	(R)VGAGAPVYmAAVLEYLTAEILELAGNAAR(D)	1475.27	2	2949.54	70.4	0.00162

	Accession Number	Protein(s) inferred	Mol Wt (kDa)	Number of Unique Peptides	Sequence Coverage	Position in sequence	Peptide Sequence	Precursor m/z	Charge observed	Calculated mass (M+H)	Mascot score	Mascot Expect value
110	Q17QC8	tr|Q17QC8|Complement factor properdin	50	1	2.80%	102 - 114	(R)HcTGWGEQcFPEK(V)	545.89	3	1635.67	32.4	0.379
						102 - 114	(R)HcTGWGEQcFPEK(V)	545.89	3	1635.67	38.2	0.102
112	Q02878	sp|Q02878|60S ribosomal protein L6	32	2	11.10%	142 - 156	(R)ASITPGTILIILTGR(H)	763.47	2	1525.93	59.3	0.00435
						167 - 183	(K)QLASGLLLVTGPLVLNR(V)	882.54	2	1764.08	58.8	0.00269
113	P19034	sp|P19034|Apolipoprotein C-II	11	1	23.80%	78 - 101	(K)STAAVTTYAGIITDQVFSVLSGKD(-)	1222.63	2	2444.26	65.8	0.00536
114	P05388	sp|P05388|60S acidic ribosomal protein P0	34	1	4.10%	150 - 162	(R)GTIEILSDVQLIK(T)	714.92	2	1428.83	49.5	0.132
115	P81187	sp|P81187|Complement factor B	85	1	2.23%	680 - 696	(R)FLcTGGVDPYADPNTcK(G)	957.92	2	1914.84	55.1	0.00792
116	P09651	sp|P09651|Heterogeneous nuclear ribonucleoprotein A1	38	1	4.30%	16 - 31	(K)LFIGGLSFETTDESLR(S)	892.96	2	1784.91	81.2	0.00012
117	P34955	sp|P34955|Alpha-1-antiproteinase	46	2	7.69%	197 - 213	(K)VLDPNTVFALVNYISFK(G)	647.36	3	1940.05	52.5	0.0811
						323 - 337	(K)SVLGDVGITEVFSDR(A)	797.41	2	1593.81	54.5	0.0629
118	Q28085	sp|Q28085|Complement factor H	140	1	1.13%	841 - 854	(K)ENYLIQDAEEIVcK(D)	862.41	2	1723.82	87.0	2.1E-05
119	Q0VCM5	sp|Q0VCM5|Inter-alpha-trypsin inhibitor heavy chain H1	101	1	1.43%	560 - 572	(R)LWAYLTIQELLAK(R)	781.45	2	1561.90	50.9	0.0757
120	P20930	sp|P20930|Filaggrin	435	1	0.37%	2 - 16	(M)sTLLENIFAIINLFK(Q)	889.51	2	1778.01	58.7	0.0135
						2 - 16	(M)sTLLENIFAIINLFK(Q)	889.51	2	1778.01	47.3	0.186

	Accession Number	Protein(s) inferred	Mol Wt (kDa)	Number of Unique Peptides	Sequence Coverage	Position in sequence	Peptide Sequence	Precursor m/z	Charge observed	Calculated mass (M+H)	Mascot score	Mascot Expect value
121	NP_001159957	ref|NP_001159957|Complement component 4A	192	2	10.90%	728 - 741	(R)KPFLSccQFAESLR(K)	581.62	3	1742.84	42.3	0.629
						81 - 95	(R)DFILLNVPIPQAQAR(I)	847.98	2	1694.96	55.9	0.0269
122	O00159	sp|O00159|Myosin-Ic	121	2	3.39%	606 - 618	(K)MSLLQLVEILQSK(E)	751.44	2	1501.87	57.9	0.0186
						606 - 618	(K)mSLLQLVEILQSK(E)	759.43	2	1517.86	95.2	3.9E-06
						46 - 68	(R)VGVQDFVLLENFTSEAAFIENLR(R)	1306.17	2	2611.34	69.1	0.00229
123	Q3T052	sp|Q3T052|Inter-alpha-trypsin inhibitor heavy chain H4	101	2	3.49%	154 - 163	(R)HLGAYELLLK(V)	578.84	2	1156.67	44.6	0.397
						548 - 569	(R)LWAYLTIQQLLEQMVSALDAEK(Q)	1282.18	2	2563.35	60.1	0.0182
124	P56652	sp|P56652|Inter-alpha-trypsin inhibitor heavy chain H3	99	3	6.73%	150 - 161	(K)VTFELTYEELLK(R)	742.90	2	1484.79	62.6	0.00774
						557 - 569	(R)LWAYLTIEQLLDK(R)	803.45	2	1605.89	85.8	3.9E-05
						557 - 569	(R)LWAYLTIEQLLDK(R)	803.45	2	1605.89	73.2	0.00071
						181 - 215	(K)HFEITVDIFEPQGISTLDAEASFITNDLLGSALTK(S)	1264.98	3	3792.92	63.8	0.00706
125	O75787	sp|O75787|Renin receptor	38	1	6.86%	176 - 199	(R)NNEVDLLFLSELQVLHDISSLLSR(H)	918.83	3	2754.47	73.1	0.00077
136	P68431	sp|P68431|Histone H3.1	15	1	23.50%	85 - 116	(R)FQSSAVmALQEAcEAYLVGLFEDTNLcAIHAK(R)	1201.57	3	3602.70	76.8	0.0002
127	Q2KJC7	tr|Q2KJC7|Periostin, osteoblast specific factor	86	1	9.37%	229 - 251	(R)VLTQIGTSIQDFIEAEDELSSFR(A)	1299.65	2	2598.29	85.2	5.5E-05

	Accession Number	Protein(s) inferred	Mol Wt (kDa)	Number of Unique Peptides	Sequence Coverage	Position in sequence	Peptide Sequence	Precursor m/z	Charge observed	Calculated mass (M+H)	Mascot score	Mascot Expect value
128	Q3ZBS7	tr|Q3ZBS7|Vitronectin	53	3	9.24%	173 - 182	(R)GLYcYELDEK(A)	645.29	2	1289.57	48.5	0.0489
						194 - 208	(R)DVWGIEGPIDAAFTR(V)	823.91	2	1646.82	60.0	0.0141
						315 - 333	(R)LLFWGGSYGGAGQPQLISR(N)	1004.03	2	2007.05	61.8	0.0129
129	O75531	sp|O75531|Barrier-to-autointegration factor	10	1	13.50%	42 - 53	(K)AYVVLGQFLVLK(K)	675.41	2	1349.82	67.2	0.00063
130	Q02809	sp|Q02809|Procollagen-lysine,2-oxoglutarate 5-dioxygenase 1	83	1	2.06%	206 - 220	(R)IFQNLDGALDEVVLK(F)	837.46	2	1673.91	92.7	9.5E-06
131	P54709	sp|P54709|Sodium/potassium-transporting ATPase subunit beta-3	31	1	5.02%	18 - 31	(K)LFIYNPTTGEFLGR(T)	814.43	2	1627.85	56.5	0.0346
132	P04815	sp|P04815|Spleen trypsin inhibitor I	10	1	13.00%	62 - 74	(K)AGFcETFVYGGcK(A)	748.32	2	1495.64	83.0	8.5E-06
