# Supplementary material for: Combination of hydrogel nanoparticles and proteomics to reveal secreted proteins associated with decidualization of human uterine stromal cells
Source: Proteome Sci. 2011 Sep 1;9:50. doi: 10.1186/1477-5956-9-50 (PMC3184050; doi:10.1186/1477-5956-9-50)
Supplement: Additional file 3 — Table S3. Secretome proteins captured by SEAN and identified by mass spectrometry that met the inclusion criteria. The numbers listed in rows indicate the number of distinct peptides identified by mass spectrometry from the media of decidualized cells. [file 1477-5956-9-50-S3.PDF]

Additional file 3. Secreteome proteins captured by SEAN and identified by mass spectroscopy. 53 proteins were deemed most relevent to our study. Scaffold was used to interrogate and analysed the data. All proteins identified by one peptide were manually validated

	Accession Number	Protein(s) inferred	Mol Wt (kDa)	Number of Unique Peptides	Sequence Coverage	Position in sequence	Peptide Sequence	Precursor m/z	Charge observed	Calculated mass (M+H)	Mascot score	Mascot Expect value	localization [pubmed]
1	Q14204	sp|Q14204|Cytoplasmic dynein 1 heavy chain 1	532	2	0.72%	2899 - 2921	(K)VFYEEELDVPLVLFNEVLDHVLR(I)	929.83	3	2787.46	47.7	0.315	C
							(K)VDDLLIIEEK(I)	593.83	2	1186.66	50.1	0.169	
2	P04406	sp|P04406|Glyceraldehyde-3-phosphate dehydrogenase	36	2	10.40%	310 - 323	(K)LISWYDNEFGYSNR(V)	882.40	2	1763.80	65.5	0.00151	S
						87 - 107	(K)WGDAGAEYVVESTGVFTTmEK(A)	1147.02	2	2293.03	72.1	0.0003	
3	Q9BRX8	sp|Q9BRX8|Uncharacterized protein C10orf58	25	1	4.80%	207 - 217	(K)VNLLSVLEAAK(M)	578.85	2	1156.69	62.6	0.00561	S
4	P07942	sp|P07942|Laminin subunit beta-1	198	1	1.23%	1509 - 1530	(R)NFLTQDSADLDSIEAVANEVLK(M)	1196.60	2	2392.19	91.1	1.4E-05	S

	Accession Number	Protein(s) inferred	Mol Wt (kDa)	Number of Unique Peptides	Sequence Coverage	Position in sequence	Peptide Sequence	Precursor m/z	Charge observed	Calculated mass (M+H)	Mascot score	Mascot Expect value	localization [pubmed]
5	P07355	sp|P07355|Annexin A2	38	3	10.90%	314 - 324	(K)SLYYYIQQDTK(G)	711.35	2	1421.70	47.5	0.218	S
						234 - 245	(K)SYSPYDMLESIR(K)	730.84	2	1460.67	45.5	0.162	
						50 - 63	(K)GVDEVTIVNILTNR(S)	771.93	2	1542.85	71.6	0.00109	
6	P14618	sp|P14618|Pyruvate kinase isozymes M1/M2 Tax	57	3	9.60%	174 - 186	(K)IYVDDGLISLQVK(Q)	731.91	2	1462.82	63.1	0.00723	C
						231 - 246	(K)FGVEQDVDmVFASFIR(K)	938.45	2	1875.90	69.7	0.00138	
						401 - 422	(R)LAPITSDPTEATAVGAVEASFK(C)	1088.06	2	2175.12	73.9	0.00077	
7	P06733	sp|P06733|Alpha-enolase	47	1	5.07%	373 - 394	(R)SGETEDTFIADLVVGLcTGQIK(T)	1177.08	2	2353.16	63.6	0.00723	C
8	P61978	sp|P61978|Heterogeneous nuclear ribonucleoprotein K	50	2	6.05%	208 - 219	(K)IILDLISESPIK(G)	670.91	2	1340.80	64.4	0.00256	C
						87 - 102	(R)ILSISADIETIGEILK(K)	858.00	2	1714.98	81.2	7.9E-05	
9	Q29963	sp|Q29963|HLA class I histocompatibility antigen, Cw-6 alpha chain	40	1	3.83%	46 - 59	(R)FISVGYVDDTQFVR(F)	823.41	2	1645.82	68.8	0.0019	S

	Accession Number	Protein(s) inferred	Mol Wt (kDa)	Number of Unique Peptides	Sequence Coverage	Position in sequence	Peptide Sequence	Precursor m/z	Charge observed	Calculated mass (M+H)	Mascot score	Mascot Expect value	localization [pubmed]
10	Q99623	sp|Q99623|Prohibitin-2	33	1	5.69%	55 - 71	(R)IGGVQQDTILAEGLHFR(I)	618.67	3	1853.99	65.0	0.00524	M
11	P14625	sp|P14625|Endoplasmin	92	1	1.99%	709 - 724	(K)TVLDLAVVLFETATLR(S)	881.01	2	1761.02	51.2	0.0629	S
12	Q16270	sp|Q16270|Insulin-like growth factor-binding protein 7	29	4	19.90%	79 - 89	(R)GYcAPGMEcVK(S)	636.26	2	1271.52	36.2	0.262	S
						263 - 275	(K)ITVVDALHEIPVK(K)	717.42	2	1433.84	62.8	0.00446	
						154 - 167	(K)GTcEQGPSIVTPPK(D)	735.87	2	1470.73	55.3	0.0354	
						117 - 134	(R)YPVcGSDGTTYPSGcQLR(A)	1009.44	2	2017.88	83.5	9.8E-06	
13	P84243	sp|P84243|Histone H3.3	15	1	23.50%	85 - 116	(R)FQSAAIGALQEASEAYLVGLFEDTNLcAIHAK(R)	1146.57	3	3437.71	66.7	0.00354	N

	Accession Number	Protein(s) inferred	Mol Wt (kDa)	Number of Unique Peptides	Sequence Coverage	Position in sequence	Peptide Sequence	Precursor m/z	Charge observed	Calculated mass (M+H)	Mascot score	Mascot Expect value	localization [pubmed]
14	Q15113	sp|Q15113|Procollagen C-endopeptidase enhancer 1	47	5	23.60%	395 - 408	(K)GVSYLLmGQVEENR(G)	805.90	2	1610.78	69.7	0.00129	S
						92 - 107	(R)YDALEVFAGSGTSGQR(L)	829.39	2	1657.78	89.8	8.7E-06	
						235 - 271	(K)FcGDAVPGSISSEGNELLVQFVSDLSVTADGFSASYK(T)	964.21	4	3853.81	51.1	0.0757	
						43 - 61	(K)GESGYVASEGFPNLYPPNK(E)	1013.48	2	2025.96	58.2	0.0151	
						325 - 344	(R)TGTLQSNFcASSLVVTATVK(S)	1042.54	2	2084.07	109.0	2.2E-07	
15	P01033	sp|P01033|Metalloproteinase inhibitor 1	23	1	5.80%	71 - 82	(K)GFQALGDAADIR(F)	617.32	2	1233.62	52.5	0.0849	S
16	P50454	sp|P50454|Serpin H1	46	2	9.81%	134 - 148	(R)LYGPSSVSFADDFVR(S)	830.40	2	1659.80	48.8	0.141	S
						61 - 86	(K)DQAVENILVSPVVVASSLGLVSLGGK(A)	1276.22	2	2551.43	45.4	0.223	

	Accession Number	Protein(s) inferred	Mol Wt (kDa)	Number of Unique Peptides	Sequence Coverage	Position in sequence	Peptide Sequence	Precursor m/z	Charge observed	Calculated mass (M+H)	Mascot score	Mascot Expect value	localization [pubmed]
17	P08670	sp|P08670|Vimentin	53	1	2.15%	130 - 139	(K)ILLAELEQLK(G)	585.36	2	1169.71	54.7	0.0208	C
18	P08254	sp|P08254|Stromelysin-1	53	3	6.08%	118 - 127	(R)IVNYTPDLPK(D)	580.32	2	1159.64	51.1	0.144	S
						79 - 88	(K)LDSDTLEVmR(K)	597.79	2	1194.57	65.4	0.00256	
						37 - 45	(K)YLENYYDLK(K)	610.79	2	1220.58	44.9	0.315	
19	Q92626	sp|Q92626|Peroxidasin homolog	165	3	3.58%	653 - 662	(R)SPNDLLALFR(Y)	573.32	2	1145.63	76.0	0.00048	S
						619 - 637	(R)NGDPFVATSIV EAIATVDR(A)	988.50	2	1975.01	76.8	0.00037	
						1204 - 1227	(R)LYGSTLNIDLFP ALVVEDLVPGSR(L	1294.70	2	2588.40	77.4	0.00029	
20	Q07020	sp|Q07020|60S ribosomal protein L18	21	1	6.91%	120 - 132	(K)ILTFDQLALDSP K(G)	730.90	2	1460.80	67.3	0.0033	M

	Accession Number	Protein(s) inferred	Mol Wt (kDa)	Number of Unique Peptides	Sequence Coverage	Position in sequence	Peptide Sequence	Precursor m/z	Charge observed	Calculated mass (M+H)	Mascot score	Mascot Expect value	localization [pubmed]
21	P07437	sp|P07437|Tubulin beta chain	49	6	20.50%	310 - 318	(R)YLTVAAVFR(G)	520.30	2	1039.59	53.9	0.0308	C
						381 - 390	(R)ISEQFTAmFR(R)	623.30	2	1245.59	68.9	0.00098	
						47 - 58	(R)ISVYYNEATGGK(Y)	651.32	2	1301.64	50.4	0.0849	
						104 - 121	(K)GHYTEGAELVDSVLDVVR(K)	653.67	3	1958.98	48.4	0.234	
						217 - 241	(K)LTTPTYGDLNHLVSATMSGVTTcLR(F)	903.45	3	2708.34	47.5	0.301	
						217 - 241	(K)LTTPTYGDLNHLVSATmSGVTTcLR(F)	908.78	3	2724.33	48.0	0.239	
						363 - 379	(K)MAVTFIGNSTAIQELFK(R)	935.49	2	1869.98	75.6	0.00048	
						363 - 379	(K)mAVTFIGNSTAIQELFK(R)	943.49	2	1885.97	91.0	1.4E-05	
22	P35579	sp|P35579|Myosin-9	226	1	0.71%	342 - 355	(R)VISGVLQLGNIVFK(K)	743.95	2	1486.90	63.7	0.00199	C
23	P35442	sp|P35442|Thrombospondin-2	129	1	1.71%	204 - 223	(R)GLLQNVHLVFENSVEDILSK(K)	752.07	3	2254.21	55.5	0.0467	S
24	B3KQX9	tr|B3KQX9|Protein Wnt	32	1	3.44%	162 - 171	(K)TcWLQLADFR(K)	655.32	2	1309.64	56.7	0.0251	S

	Accession Number	Protein(s) inferred	Mol Wt (kDa)	Number of Unique Peptides	Sequence Coverage	Position in sequence	Peptide Sequence	Precursor m/z	Charge observed	Calculated mass (M+H)	Mascot score	Mascot Expect value	localization [pubmed]
25	Q08431	sp|Q08431|Lactadherin	43	4	12.70%	298 - 308	(K)EVTGIITQGAR(N)	572.82	2	1144.63	76.3	0.00042	S
						356 - 365	(K)NLFETPILAR(Y)	587.33	2	1173.66	46.8	0.371	
						136 - 148	(R)MWVTGVVTQGASR(L)	696.36	2	1391.71	65.7	0.00456	
						93 - 107	(R)VTFLGLQHWVPELAR(L)	883.49	2	1765.98	45.5	0.362	
26	P52823	sp|P52823|Stanniocalcin-1	27	3	15.00%	34 - 44	(R)VAAQNSAEVVR(C)	572.31	2	1143.61	65.7	0.00388	S
						120 - 131	(R)MIAEVQEEcYSK(L)	743.83	2	1486.66	75.4	0.00012	
						166 - 179	(R)SLLEcDEDTVSTIR(D)	819.39	2	1637.77	77.0	0.00022	
27	P60660	sp|P60660|Myosin light polypeptide 6	16	2	13.90%	14 - 21	(K)EAFQLFDR(T)	513.26	2	1025.51	49.4	0.115	C
						38 - 50	(R)ALGQNPTNAEVLK(V)	677.87	2	1354.73	53.8	0.0536	
28	Q9Y6B6	sp|Q9Y6B6|GTP-binding protein SAR1b	22	1	5.56%	28 - 38	(K)LVFLGLDNAGK(T)	573.83	2	1146.65	62.5	0.00774	S

	Accession Number	Protein(s) inferred	Mol Wt (kDa)	Number of Unique Peptides	Sequence Coverage	Position in sequence	Peptide Sequence	Precursor m/z	Charge observed	Calculated mass (M+H)	Mascot score	Mascot Expect value	localization [pubmed]
29	P43307	sp|P43307|Translocon-associated protein subunit alpha	32	1	2.80%	103 - 110	(K)FLVGFTNK(G)	463.26	2	925.51	51.1	0.0574	S
30	P35555	sp|P35555|Fibrillin-1	312	1	0.45%	962 - 974	(R)YEDEEcTLPIAGR(H)	776.85	2	1552.70	50.9	0.0426	S
31	P03956	sp|P03956|Interstitial collagenase	53	1	2.77%	376 - 388	(K)HIDAALSEENTGK(T)	692.84	2	1384.67	63.2	0.00489	S
32	P00750	sp|P00750|Tissue-type plasminogen activator	62	2	5.16%	125 - 136	(R)ATcYEDQGISYR(G)	731.82	2	1462.63	49.4	0.0269	S
						397 - 413	(K)EFDDDTYDNDIALLQLK(S)	1014.47	2	2027.95	64.2	0.00294	
33	P04083	sp|P04083|Annexin A1	38	2	7.80%	114 - 124	(K)TPAQFDADELR(A)	631.80	2	1262.60	58.7	0.012	C
						189 - 204	(R)SEDFGVNEDLADSDAR(A)	870.37	2	1739.74	84.8	4.8E-06	

	Accession Number	Protein(s) inferred	Mol Wt (kDa)	Number of Unique Peptides	Sequence Coverage	Position in sequence	Peptide Sequence	Precursor m/z	Charge observed	Calculated mass (M+H)	Mascot score	Mascot Expect value	localization [pubmed]
34	Q8WUJ3	sp|Q8WUJ3|Protein KIAA1199	152	4	3.45%	798 - 807	(R)GGDVWLDScR(F)	582.76	2	1164.51	46.2	0.0706	S
						1347 - 1357	(K)IFQVVPIPVVK(K)	619.90	2	1238.79	36.8	0.397	
						231 - 242	(R)ILSVAVNDEGSR(N)	630.33	2	1259.66	63.3	0.0074	
						1171 - 1184	(K)NAGVSDcTATAYPK(F)	727.83	2	1454.66	81.6	3.6E-05	
35	Q01995	sp|Q01995|Transgelin	22	2	9.95%	100 - 108	(K)AAEDYGVIK(T)	483.25	2	965.49	56.0	0.0338	C
						162 - 172	(R)EFTESQLQEGK(H)	648.31	2	1295.61	57.2	0.0166	
36	Q92743	sp|Q92743|Serine protease HTRA1	51	4	10.00%	30 - 40	(R)SAPLAAGcPDR(C)	557.77	2	1114.53	56.8	0.0141	S
						169 - 178	(K)YNFIADVVEK(I)	599.31	2	1197.62	52.5	0.0706	
						347 - 359	(K)VTAGISFAIPSDK(I)	653.36	2	1305.71	71.6	0.00126	
						153 - 166	(R)GAcGQGQEDPNSLR(H)	744.83	2	1488.65	69.7	0.00029	
37	P63104	sp|P63104|14-3-3 protein zeta/delta	27	1	5.71%	28 - 41	(K)SVTEQGAELSNEER(N)	774.86	2	1548.71	76.0	0.00015	C
38	Q9BXX0	sp|Q9BXX0|EMILIN-2	115	1	0.95%	716 - 725	(K)LDSISGNLQR(I)	551.80	2	1102.59	61.8	0.0107	S

	Accession Number	Protein(s) inferred	Mol Wt (kDa)	Number of Unique Peptides	Sequence Coverage	Position in sequence	Peptide Sequence	Precursor m/z	Charge observed	Calculated mass (M+H)	Mascot score	Mascot Expect value	localization [pubmed]
39	P23396	sp|P23396|40S ribosomal protein S3	26	1	5.35%	28 - 40	(R)ELAEDGYSGVEVR(V)	712.34	2	1423.67	60.8	0.0069	M
40	P02743	sp|P02743|Serum amyloid P-component	25	1	7.14%	150 - 165	(K)IVLGQEQDSYGGGFDK(N)	856.91	2	1712.81	94.3	3.5E-06	S
41	P36955	sp|P36955|Pigment epithelium-derived factor	46	3	9.86%	105 - 121	(R)ALYYDLISNPDIHGTYK(D)	661.67	3	1982.99	53.0	0.0811	S
						52 - 65	(K)LAAAVSNFGYDLYR(V)	780.40	2	1559.79	60.1	0.0141	
42	Q15063	sp|Q15063|Periostin, osteoblast specific factor	86	1	9.37%	229 - 251	(R)VLTQIGTSIQDFIEAEDELSSFR(A)	1299.65	2	2598.29	85.2	5.5E-05	S
43	P11021	sp|P11021|78 kDa glucose-regulated protein	72	1	2.75%	307 - 324	(R)IEIESFYEGEDFSETLTR(A)	1083.00	2	2164.99	82.2	4E-05	C
44	P60709	sp|P60709|Actin, cytoplasmic 1	41	4	16.30%	291 - 312	(R)KDLYANTVLSGGTTmYPGIADR(M)	787.06	3	2359.16	64.7	0.00524	C
						239 - 254	(K)SYELPDGQVITIGNER(F)	895.95	2	1790.89	59.0	0.0199	
						292 - 312	(K)DLYANTVLSGGTTmYPGIADR(M)	1116.03	2	2231.07	81.0	8.7E-05	
						216 - 238	(K)LcYVALDFEQEMATAASSSSLEK(S)	1275.59	2	2550.17	98.0	1.1E-06	
45	P21589	sp|P21589|5'-nucleotidase	63	1	2.61%	148 - 162	(K)GPLASQISGLYLPYK(V)	803.95	2	1606.88	61.8	0.0104	S

	Accession Number	Protein(s) inferred	Mol Wt (kDa)	Number of Unique Peptides	Sequence Coverage	Position in sequence	Peptide Sequence	Precursor m/z	Charge observed	Calculated mass (M+H)	Mascot score	Mascot Expect value	localization [pubmed]
46	P21796	sp|P21796|Voltage-dependent anion-selective channel protein 1	30	2	8.13%	257 - 266	(K)LTLSALLDGK(N)	515.81	2	1030.62	67.0	0.00288	C
						97 - 109	(K)LTFDSSFSPNTGK(K)	700.84	2	1400.67	58.8	0.0132	
47	P07737	sp|P07737|Profilin-1	15	1	10.00%	92 - 105	(K)STGGAPTFNVTVTK(T)	690.37	2	1379.72	62.0	0.0107	C
48	P50914	sp|P50914|60S ribosomal protein L14	23	1	5.58%	24 - 35	(K)LVAIVDVIDQNR(A)	677.89	2	1354.77	63.5	0.00406	N
49	P10412	sp|P10412|Histone H1.4	21	1	4.11%	55 - 63	(R)SGVSLAALK(K)	423.26	2	845.51	62.3	0.00869	N
50	P12814	sp|P12814|Alpha-actinin-1	103	3	4.37%	727 - 738	(R)TINEVENQILTR(D)	715.39	2	1429.77	57.9	0.0275	C
						48 - 60	(K)AGTQIENIEEDFR(D)	761.36	2	1521.72	54.7	0.0301	
						134 - 147	(R)FAIQDISVEETSAK(E)	769.39	2	1537.78	63.2	0.00757	
51	P22626	sp|P22626|Heterogeneous nuclear ribonucleoproteins A2/B1	37	1	2.83%	138 - 147	(K)IDTIEIITDR(Q)	594.83	2	1188.65	89.8	2E-05	N
52	P25705	sp|P25705|ATP synthase subunit alpha, mitochondrial	59	1	2.71%	59 - 73	(R)ILGADTSVDLEETGR(V)	788.40	2	1575.79	74.0	0.00057	M
